# Microbiome-Directed Bioactive Strategies in Skin Aging: Mechanistic Insights and Precision Nanocarrier Delivery Approaches

**DOI:** 10.3390/molecules31152563

**Published:** 2026-07-23

**Authors:** Beatrice Bălăceanu-Gurău, Ina Sotiri, Patricija Girštautė, Anastasia Patricia Șmocot-Stănescu, Iulia-Alexandra Voinea, Andreea-Laura Bărbulescu, Andrea Cortese

**Affiliations:** 1 Ph.D. Doctoral School, “Carol Davila” University of Medicine and Pharmacy, 050474 Bucharest, Romania; anastasia.patricia.stanescu@gmail.com (A.P.Ș.-S.); iulia-alexandra.voinea@drd.umfcd.ro (I.-A.V.); 2 Dermatology Department, University Hospital Centre “Mother Teresa”, 1000 Tirana, Albania; inaa.sotiri@gmail.com; 3 Center of Dermatovenereology, Vilnius University Hospital Santaros Klinikos, 08406 Vilnius, Lithuania; patricija.simkunaite@gmail.com; 4 Medlife Clinic, 011364 Bucharest, Romania; 5 “C.I. Parhon” National Institute of Endocrinology, 011863 Bucharest, Romania; 6 New Line Clinic, 500009 Brașov, Romania; laurabarbulescu@gmail.com; 7 Dermatology Unit, Policlinico San Pietro, 24036 Bergamo, Italy

**Keywords:** skin aging, skin microbiome, endocrine–microbiome–skin axis, bioactive compounds, postbiotics, phytoestrogens, antioxidants, nanocarriers, cosmeceuticals, precision dermatology

## Abstract

Skin aging is a multidimensional biological process driven by intrinsic chronological changes, exposomal stress, endocrine–metabolic shifts, extracellular matrix remodeling, inflammaging, oxidative injury, barrier impairment, and microbiome dysbiosis. This review integrates current evidence on the endocrine–microbiome–skin axis and evaluates microbiome-directed bioactive strategies for preserving cutaneous homeostasis during aging. Particular attention is given to probiotics, prebiotics, postbiotics, synbiotics, phytoestrogens, polyphenols, bioactive peptides, antioxidants, mitochondrial protectors, adaptogens, and metabolic modulators. Their mechanisms are discussed in relation to collagen homeostasis, mitochondrial function, lipid barrier integrity, immune regulation, microbial metabolite signaling, and systemic endocrine–metabolic status. The review also examines advanced delivery platforms, including liposomes, solid lipid nanoparticles, nanostructured lipid carriers, polymeric nanocarriers, nanoemulsions, encapsulated microbiome-active systems, and stimuli-responsive carriers, emphasizing their potential to improve compound stability, skin retention, controlled release, and target-site precision. Translational limitations are critically addressed, including strain and formulation specificity, insufficient long-term safety data, incomplete nanocarrier toxicology, regulatory ambiguity, and the need for personalization according to hormonal, metabolic, and microbiome profiles. Overall, microbiome-directed bioactive compounds combined with precision delivery systems represent a promising, but still evolving, strategy for delaying skin aging and restoring cutaneous homeostasis.

## 1. Introduction

### 1.1. Skin Aging as a Multidimensional Biological Process

Skin aging is a progressive and multifactorial biological process resulting from the interaction between intrinsic chronological mechanisms and extrinsic environmental exposures. Intrinsic aging reflects genetically and metabolically programmed changes, including reduced epidermal turnover, dermal thinning, impaired fibroblast activity, altered immune surveillance, and diminished regenerative capacity, whereas extrinsic aging is mainly driven by exposomal stressors such as ultraviolet (UV) radiation, air pollution, smoking, diet, sleep disruption, and other lifestyle-related factors [1,2,3]. Although intrinsic and extrinsic aging are often described separately, they converge on overlapping molecular pathways that impair skin structure, barrier function, pigmentation homeostasis, and tissue repair [1,2,3].

At the molecular level, aging skin accumulates cellular and extracellular damage. Reactive oxygen species (ROS), generated through mitochondrial metabolism and amplified by UV radiation and environmental pollutants, induce oxidative injury to deoxyribonucleic acid (DNA), lipids, and proteins [1,4,5]. This oxidative burden contributes to mitochondrial dysfunction, telomere shortening, epigenetic alterations, impaired proteostasis, cellular senescence, and the development of a senescence-associated secretory phenotype that further disrupts tissue homeostasis [1,4,5]. These mechanisms place oxidative stress and senescence at the center of both chronological aging and photoaging [1,4,5].

A major visible consequence of these molecular changes is progressive remodeling of the dermal extracellular matrix (ECM) [2,6,7]. Aged skin shows reduced synthesis of collagen types I and III, altered elastin organization, decreased fibroblast responsiveness, and increased degradation of structural matrix components [2,6,7]. Matrix metalloproteinases (MMPs), especially MMP-1, MMP-3, and MMP-9, are induced by ROS, UV radiation, inflammatory mediators, and activation of activator protein 1 (AP-1) and nuclear factor kappa B (NF-κB), leading to dermal thinning, loss of elasticity, wrinkle formation, and increased skin fragility [2,6,7].

Chronic low-grade inflammation, or inflammaging, further amplifies these structural and functional alterations. In aged skin, persistent immune activation is promoted by oxidative stress, senescent cell accumulation, impaired barrier function, and altered communication between keratinocytes, fibroblasts, immune cells, and resident microorganisms [1,8,9]. This inflammatory microenvironment accelerates ECM degradation, compromises epidermal differentiation, delays wound healing, and increases susceptibility to inflammatory and infectious skin disorders [1,8,9].

The skin microbiome is increasingly recognized as an active component of the aging network. Age-related changes in sebum production, hydration, pH, lipid composition, immune tone, and barrier integrity reshape cutaneous microbial ecology, while dysbiosis may reinforce oxidative stress, inflammation, barrier dysfunction, and ECM degradation through altered microbial metabolites and reduced commensal-derived protective signals [10,11,12]. Therefore, skin aging should be understood not only as a cellular and structural process, but also as an ecological disturbance involving the host–microbiome interface [10,11,12].

This multidimensional view has important therapeutic implications. Because oxidative stress, inflammaging, ECM degradation, barrier impairment, and microbial dysbiosis are interconnected, effective anti-aging strategies should ideally target multiple pathways rather than a single mechanism [13,14,15]. Bioactive compounds with antioxidant, anti-inflammatory, barrier-restoring, antiglycation, and microbiome-modulating properties are therefore of growing interest, particularly when combined with advanced delivery systems designed to improve stability, skin penetration, bioavailability, and site-specific activity within the aging skin microenvironment [13,14,15].

### 1.2. Endocrine and Metabolic Contributions to Skin Aging

Beyond intrinsic and extrinsic molecular damage, skin aging is strongly influenced by endocrine and metabolic regulation. The skin is a hormonally responsive organ in which keratinocytes, fibroblasts, melanocytes, sebocytes, endothelial cells, and immune cells express hormone receptors and participate in local hormone metabolism. Estrogens, androgens, insulin-like growth factor-1 (IGF-1), glucocorticoids, thyroid hormones, and insulin-related metabolic pathways contribute to epidermal renewal, dermal matrix integrity, sebaceous activity, barrier function, immune regulation, and oxidative balance.

Estrogen decline is one of the most relevant endocrine mechanisms involved in age-related skin deterioration. Estrogens act mainly through estrogen receptor alpha (ERα) and estrogen receptor beta (ERβ), with ERβ considered the predominant estrogen receptor in human skin [16,17,18,19]. Through these pathways, estrogens support collagen synthesis, ECM turnover, vascularization, epidermal renewal, hydration, and tissue repair [16,17,18,19]. During perimenopause and menopause, declining estrogen levels and altered receptor responsiveness reduce matrix homeostasis and contribute to dermal thinning, dryness, reduced elasticity, and impaired repair [16,17,18,19].

In the early postmenopausal period, collagen content may decrease substantially, partly because fibroblast activity declines while matrix metalloproteinase activity increases [16,17,18,19]. Although the skin can locally convert dehydroepiandrosterone (DHEA) into estrogens, this compensatory pathway becomes progressively less efficient with age as circulating precursors decline and receptor expression is reduced [18,19,20]. Hormone therapy may partially improve skin hydration and elasticity, but it is not recommended solely for the treatment of skin aging [17,19].

Androgens contribute to cutaneous aging mainly through regulation of sebaceous gland biology and skin surface lipid composition. Sebocyte proliferation and lipid synthesis are stimulated by testosterone and dihydrotestosterone through androgen receptors expressed on sebocytes [21,22]. With aging, sebum production declines and lipid composition changes, which may weaken barrier support, modify microbial nutrient availability, and promote a more pro-oxidative and pro-inflammatory skin microenvironment, particularly in sebum-rich skin [21,22].

IGF-1 is another important regulator of dermal homeostasis. Activation of the IGF-1 receptor stimulates phosphoinositide 3-kinase/protein kinase B (PI3K/AKT) and mitogen-activated protein kinase (MAPK) signaling, supporting fibroblast survival, collagen synthesis, and balanced matrix remodeling [18,23,24]. However, IGF-1 signaling is context-dependent: physiological activity supports repair and structural integrity, whereas dysregulation of the IGF-1/mechanistic target of rapamycin (mTOR) axis may promote oxidative stress, cellular senescence, and impaired autophagy [23,25]. Lower circulating IGF-1 levels during aging have been associated with reduced collagen production, impaired fibroblast function, and weakened responses to UV-induced damage [23,24].

Glucocorticoid signaling links psychological stress, systemic endocrine regulation, and local skin aging mechanisms. Cortisol acts through the central hypothalamic–pituitary–adrenal (HPA) axis, while the skin also possesses a peripheral HPA-like system capable of producing and responding to stress-related mediators [26,27]. Excessive systemic or topical glucocorticoid exposure may reduce keratinocyte proliferation, suppress stratum corneum lipid synthesis, decrease collagen production, and downregulate barrier proteins such as filaggrin, involucrin, and loricrin [26,27]. Chronic stress may additionally impair epidermal stem cell renewal, delay wound healing, and amplify inflammatory and oxidative signaling [28,29]. These effects highlight the interdependence between glucocorticoid homeostasis, barrier integrity, and cutaneous immune regulation [26,30].

Thyroid hormones further influence skin biology through nuclear receptor-mediated transcriptional regulation. They modulate epidermal turnover, mitochondrial activity, thermoregulation, and hair follicle cycling, explaining the cutaneous manifestations of thyroid dysfunction, including xerosis and impaired wound healing in hypothyroidism or increased epidermal turnover, blood flow, and sweating in hyperthyroidism [31,32,33]. Although thyroid hormones are not primary anti-aging targets, their role in epidermal renewal and mitochondrial function makes them relevant systemic modulators of skin homeostasis [31,32,33].

Insulin resistance and chronic hyperglycemia directly connect systemic aging, inflammation, and structural skin deterioration [34,35,36,37]. Insulin resistance promotes low-grade systemic inflammation, while chronic hyperglycemia favors the formation of advanced glycation end products (AGEs) that accumulate in long-lived dermal proteins, particularly collagen, leading to collagen stiffening, reduced matrix flexibility, oxidative stress, and NF-κB-driven inflammatory signaling [34,35,36,37]. Together, endocrine and metabolic pathways support the concept that skin aging reflects not only local tissue damage, but also systemic hormonal and metabolic status [34,35,36,37].

Therefore, this review aims to integrate current evidence on the molecular, endocrine–metabolic, and microbiome-related mechanisms of skin aging with emerging bioactive and nanocarrier-based intervention strategies. Particular emphasis is placed on multidimensional compounds, including antioxidants, anti-inflammatory agents, microbiome modulators, phytoestrogens, peptides, postbiotics, and metabolic regulators, as well as on advanced delivery platforms designed to improve stability, skin retention, controlled release, and site-specific activity. By linking mechanistic aging pathways with precision delivery approaches, this review highlights the potential of microbiome-directed and bioactive nanotechnology as an integrated strategy for preserving cutaneous homeostasis and delaying age-associated skin decline.

Accordingly, the following section examines how endocrine and metabolic regulation interacts with the skin microbiome to shape the biological context in which these aging pathways emerge and can potentially be modified.

### 1.3. Scope and Novelty of the Present Review

Recent reviews have separately examined the relationship between the skin microbiome and barrier aging, the microbiome–wrinkle axis, microbiome-modulating bioactive compounds, conventional anti-aging active ingredients, or nanoformulated phytochemicals for skin applications [10,11,12,13,14,15]. These contributions have led to an advanced understanding of individual components of skin aging; however, they generally address endocrine regulation, microbiome modulation, bioactive compounds, and delivery technologies as partially independent topics.

The knowledge gap addressed by the present review is the lack of an integrated framework linking endocrine–metabolic changes, microbiome dynamics, molecular aging pathways, multifunctional bioactives, and precision delivery systems. In particular, this review examines how hormonal status, metabolic dysfunction, sebaceous and barrier biology, microbial metabolites, oxidative stress, inflammaging, and ECM remodeling interact within the endocrine–microbiome–skin axis. It further evaluates probiotics, postbiotics, phytoestrogens, polyphenols, peptides, antioxidants, mitochondrial protectors, and metabolic modulators according to their mechanistic targets and level of supporting evidence.

A further distinctive feature is the translation of this mechanistic framework into a layer-specific delivery perspective. Rather than considering nanocarriers solely according to carrier type, this review discusses carrier–cargo matching in relation to the intended skin compartment, including the skin surface and stratum corneum, follicular–pilosebaceous unit, viable epidermis, and potentially accessible superficial dermis. This approach explicitly addresses the epidermis–dermis penetration gap and distinguishes improved formulation performance from clinically demonstrated anti-aging benefit. Finally, the review identifies practical requirements for future implementation, including strain- and postbiotic-level characterization, carrier optimization, standardized penetration and safety assessment, and harmonized clinical endpoints.

Therefore, the present work aims to provide a mechanism-oriented and translationally focused framework for microbiome-directed precision strategies in skin aging, rather than a descriptive overview of individual active ingredients or delivery platforms alone.

## 2. The Endocrine–Microbiome–Skin Axis

The endocrine–microbiome–skin axis describes the bidirectional interactions through which hormonal signals shape the cutaneous microenvironment, while resident microorganisms and their metabolites influence barrier integrity, immune regulation, oxidative balance, and tissue repair [10,12,38,39,40,41]. In aging skin, endocrine changes affecting ECM homeostasis, sebaceous lipid production, epidermal turnover, vascularization, and inflammation may reshape microbial niches and promote dysbiosis, while microbiome-derived metabolites may further modulate inflammation, oxidative stress, and barrier function [16,17,38,42,43,44,45,46,47,48,49,50].

### 2.1. Hormonal Regulation of Skin Structure

Hormonal signaling maintains skin structure by regulating dermal matrix remodeling, sebaceous gland activity, fibroblast function, barrier integrity, and immune responses [16,17,38,42,43,44,45,46,47,48,49]. These processes also influence lipid availability, hydration, pH, antimicrobial defense, and inflammatory tone, thereby shaping the composition and function of the cutaneous microbiome [38,39,40,41,44,45,46,49].

#### 2.1.1. Estrogen-Mediated Collagen Synthesis and Dermal Vascularization

Estrogen is an important regulator of dermal ECM homeostasis, supporting collagen synthesis, remodeling, vascularization, and the structural integrity of the skin [42]. Adequate estrogen levels, particularly 17β-estradiol, are therefore essential for maintaining dermal strength and functional performance [42].

The age-related decline in DHEA reduces the skin’s capacity to sustain adequate estrogen levels, thereby contributing to estrogen deficiency after menopause [16]. As proposed by Merzel Šabović et al., menopausal skin alterations are primarily driven by systemic estrogen decline, diminished local estrogen synthesis within the skin, and decreased expression of cutaneous estrogen receptors [43]. This hypoestrogenic state contributes to dermal thinning and atrophy, reduced collagen content, decreased elasticity, and impaired vascularization [17,42,43]. Reduced vascularity may further impair wound healing and weaken the protective skin barrier [43]. Through these effects on matrix organization, vascular support, and barrier function, estrogen decline may indirectly modify the ecological conditions that sustain cutaneous microbial homeostasis.

#### 2.1.2. Androgen Signaling and Sebaceous–Microbial Balance

Androgens influence sebaceous gland growth and differentiation, terminal hair development, epidermal barrier integrity, wound healing, and cutaneous microbiome composition [44]. Sebum production is essential for skin homeostasis and is tightly regulated by endocrine and metabolic mechanisms [44]. Sebaceous glands are involved in more than lipid secretion, as they also participate in immune regulation and influence androgen metabolism, cytokine production, and antimicrobial peptide activity [45].

Sebaceous gland secretions, particularly skin surface lipids, function as nutrient substrates for cutaneous microbial communities. Microbial enzymes metabolize triglycerides into free fatty acids (FFAs), which influence microbial adhesion and local inflammatory signaling [46]. Therefore, age-related or endocrine-driven changes in sebaceous activity may alter microbial nutrient availability, antimicrobial defense, and inflammatory tone, linking androgen signaling directly to sebaceous–microbial balance.

#### 2.1.3. IGF-1 and Dermal Fibroblast Function

The accumulation of senescent fibroblasts contributes to skin aging because these cells progressively lose their normal identity and functional capacity [50]. Fibroblast senescence also amplifies inflammation through the secretion of cytokines, chemokines, and other bioactive mediators that activate immune cells and promote local, and potentially systemic, inflammatory responses [50].

Age-related intrinsic and extrinsic factors modulate several signaling pathways in dermal fibroblasts, including nuclear factor erythroid 2-related factor 2 (NRF2), transforming growth factor beta (TGF-β), IGF-1, and mTOR cascades [47]. Available evidence supports a role for IGF-1 signaling in dermal aging, while JunB-mediated inhibition of IGF-1 gene activation lowers IGF-1 levels, reduces PI3K/AKT signaling, disrupts the skin stem cell niche, and contributes to aging progression [47,48]. Through senescence-associated inflammatory signaling, altered fibroblast activity may also influence epidermal differentiation, immune surveillance, and the host–microbial interface.

#### 2.1.4. Cortisol-Induced Barrier Disruption and Immune Modulation

Stress activates the brain–pituitary–adrenal axis and stimulates the release of corticotropin-releasing hormone (CRH), adrenocorticotropic hormone (ACTH), and glucocorticoids such as cortisol [38]. The skin also participates in this response, as keratinocytes and melanocytes can produce ACTH, while CRH may stimulate local cortisol production and reinforce glucocorticoid signaling [38]. In chronic stress, cortisol-mediated immune suppression may impair barrier function and promote a pro-inflammatory cutaneous environment [38].

Cortisol increases ROS and reduces epidermal lipids, hyaluronic acid, type I collagen, and stratum corneum hydration [49]. These changes weaken the skin barrier and increase susceptibility to irritation, dryness, infection, and delayed wound healing [49]. By disrupting barrier integrity and immune regulation, chronic stress and glucocorticoid imbalance may also create conditions that favor microbial imbalance and inflammatory amplification [49].

#### 2.1.5. Mechanistic Links Between Hormonal Signaling and Cutaneous Microbial Ecology

Hormonal regulation of the skin microbiota is likely mediated predominantly through hormone-induced changes in the host cutaneous niche rather than through direct microbial sensing of circulating hormones. The principal mechanisms include modulation of sebaceous lipid production, stratum corneum lipid organization, hydration, surface pH, antimicrobial defense, and local immune tone. These factors determine nutrient availability, microbial adhesion, competition between resident taxa, and the capacity of the skin barrier to restrict microbial overgrowth or penetration [16,17,38,39,40,41,44,45,46].

Androgen signaling provides a clear example of this host-mediated mechanism. By regulating sebocyte proliferation and lipid synthesis, androgens modify the availability and composition of triglycerides, wax esters, squalene, and other sebum-derived substrates. These lipids can be metabolized by resident microorganisms, particularly Cutibacterium species, into FFAs that influence surface acidification, microbial growth, and local inflammatory signaling [44,45,46]. Therefore, androgen-related changes in sebaceous activity may alter microbial ecology indirectly through changes in substrate availability and the physicochemical properties of the follicular microenvironment.

Estrogen decline may influence the cutaneous microbiome through a different set of mechanisms. Reduced estrogen signaling is associated with changes in epidermal turnover, hydration, collagen homeostasis, vascularization, and barrier repair, all of which may modify the ecological conditions that support commensal stability [16,17,42,43]. Similarly, glucocorticoid excess and chronic stress may impair epidermal lipid synthesis, barrier recovery, and local immune regulation, potentially altering microbial adherence and susceptibility to inflammation-associated dysbiosis [26,27,30,38,39,40,41].

The reverse arm of this interaction is also relevant at the skin surface. Microbial lipases, free fatty acids, short-chain fatty acids (SCFAs), antimicrobial compounds, and microbe-associated molecular patterns can influence keratinocyte differentiation, cytokine signaling, antimicrobial defense, and oxidative balance [40,41,50,51,52]. These microbial signals may in turn modify the hormone-responsive inflammatory and metabolic environment of the skin. However, direct evidence that skin-derived microbial metabolites regulate local estrogen, androgen, or glucocorticoid signaling in physiologically aged human skin remains limited. Accordingly, the strongest current evidence supports an indirect bidirectional loop in which hormones shape microbial niches through host barrier and lipid biology, whereas microbial products influence hormone-responsive epithelial and immune pathways.

Together, these host-mediated mechanisms show that hormonal signaling influences microbial ecology primarily by modifying the physicochemical and immunological properties of the cutaneous niche. The next section examines how aging-related changes in this niche are reflected in the composition and functional capacity of the skin microbiome.

### 2.2. Microbiome Dynamics in Aging Skin

The skin microbiome contributes to barrier defense, immune education, antimicrobial protection, and metabolic signaling [10,12,39,40,41]. During aging, changes in sebum production, hydration, pH, lipid composition, immune responsiveness, and barrier integrity reshape microbial ecology, which may in turn influence oxidative stress, inflammation, and tissue repair [10,12,39,51,52,53,54,55,56,57].

#### 2.2.1. Age-Related Microbial Shifts and Functional Changes

Skin aging is accompanied by changes in microbiome composition and function that may impair barrier function and overall skin health. Studies report reductions in homeostasis-associated commensals such as *Cutibacterium acnes* and *Staphylococcus epidermidis*, alongside increased prevalence of potentially pathogenic or inflammation-associated species such as *Staphylococcus aureus* [51]. This imbalance may weaken defense against pathogens and environmental stressors [51]. Reduced microbial diversity and functional capacity in aged skin have also been associated with impaired barrier function, increased transepidermal water loss (TEWL), and greater pro-inflammatory activity [52]. Because *C. acnes* produces antimicrobial compounds, immunomodulatory factors, and SCFAs, its age-related decline may contribute to clinical and physiological features of aged skin [53].

#### 2.2.2. Dysbiosis and Inflammatory Amplification

Dysbiosis refers to disruption of the balanced interaction between the host and resident commensal microbiota, often involving reduced microbial stability, altered bacterial diversity, and loss of protective microbial functions [12]. When this balance is disturbed, dysbiosis may increase susceptibility to inflammatory and infectious skin disorders and may contribute to accelerated skin aging [12].

Aging further exacerbates inflammaging, a chronic low-grade inflammatory state associated with microbial dysbiosis [10]. This condition contributes to impaired barrier function, increased transepidermal water loss, and greater susceptibility to inflammatory skin disorders [10]. Microbiome alterations are therefore thought to sustain the mild inflammation that develops with aging and contributes to progressive tissue deterioration, including in the skin [10].

#### 2.2.3. Oxidative Stress and Microbial Metabolite Imbalance

Oxidative stress is a key feature of aging and may be modulated by the skin microbiome. Microbial imbalance in aged skin has been associated with increased pro-oxidative enzyme activity, which may contribute to hyperpigmentation, fine lines, and reduced skin elasticity [55]. Dysbiosis may also favor microorganisms capable of increasing ROS production, leading to oxidative damage to lipids, proteins, and DNA when antioxidant capacity is exceeded [56].

Conversely, the skin microbiome may exert protective antioxidant effects. Harel et al. reported that *Sphingomonas* and *Erythrobacteraceae* may protect against UV radiation through compounds that reduce solar damage to the skin [57]. Further investigation of a *Sphingomonas* strain showed strong resistance to UV irradiation and reduced ROS levels in human keratinocytes [57]. These findings suggest that the skin microbiome may contribute to protection against UV-induced damage and support the potential of microbiome-based photoprotective strategies [55].

#### 2.2.4. Barrier Dysfunction, Lipid Remodeling, and Microbial Ecology

Age-related changes in the skin are accompanied by shifts in microbiome composition and function that may impair barrier integrity and overall skin health [39]. The skin microbiota is increasingly recognized as an essential component of the cutaneous barrier, interacting with keratinocytes, immune cells, nerves, and other barrier elements [40,41]. During aging, alterations in stratum corneum structure and lipid composition contribute to compromised barrier integrity, reduced hydration, and greater vulnerability to infection [39].

Skin lipids synthesized by epidermal cells, including cholesterol, ceramides, and FFAs, may serve as nutrient substrates for cutaneous microbial communities [54]. Age-dependent lipid remodeling has been associated with microbial shifts, including positive associations between specific ceramide species and increased *Streptococcus* abundance [54]. Comparative analyses across the forearm, buttock, and facial skin have shown age-related changes in fatty acids, ceramides, cholesterol, and sphingolipids, together with altered abundance of *Cutibacterium*, *Streptococcus*, *Corynebacterium*, *Micrococcus*, *Lactobacillus*, *Anaerococcus*, and *Finegoldia* [54]. These microbial changes were also associated with age-related alterations in sebocyte area, natural moisturizing factors, antimicrobial peptides, and skin lipid profiles, suggesting that age-related microbiome changes may reflect broader alterations in skin barrier biology, lipid metabolism, sebaceous activity, and antimicrobial defense [54].

These age-related shifts in barrier structure, lipid composition, sebaceous activity, and microbial composition are not isolated phenomena. Rather, they provide the basis for bidirectional endocrine–microbiome crosstalk, in which host hormonal and metabolic signals influence microbial ecology while microbial products may reciprocally affect local and systemic signaling pathways.

### 2.3. Bidirectional Endocrine–Microbiome Interactions in Skin Aging

The endocrine–microbiome–skin axis should be understood as a bidirectional network in which hormonal signals influence microbial composition and function, while microbial communities and their metabolites regulate endocrine, metabolic, immune, and inflammatory pathways [10,11,12,38,42,43]. Although much of the evidence concerns the gut microbiome, these systemic interactions are relevant to skin aging because gut microbial metabolism may influence circulating sex hormones, cardiometabolic risk, chronic inflammation, oxidative stress, and bioactive metabolite availability [10,11,12,38,42,43]. In aging, hormonal decline, metabolic dysfunction, and microbial dysbiosis may therefore converge to amplify inflammaging and impair skin barrier function [10,11,12,38,42,43].

#### 2.3.1. Hormonal Influence on Microbial Ecology

The relationship between sex hormones and microbial ecology is bidirectional, as estrogens and progesterone may influence microbial diversity and composition, while the gut microbiome can modulate sex hormone metabolism through enzymatic activities involved in enterohepatic circulation and estrogen deconjugation [58]. This interaction is relevant in aging because hormonal decline may reshape microbial communities, while microbiome alterations may contribute to systemic endocrine and metabolic changes [58,59,60].

Estrogen appears to regulate microbial diversity, although available evidence remains heterogeneous and may vary according to age, menopausal status, body composition, diet, and metabolic health [58]. Higher circulating levels of estrogens and progesterone have been associated with increased gut microbial diversity, whereas hormonal decline during menopause is associated with a gut microbiome profile that becomes more similar to that observed in men [58,59,60]. Peters et al. reported that menopause is associated with alterations in the gut microbiome and estrobolome, with potential implications for adverse cardiometabolic risk [60]. Thus, the menopausal transition may be considered not only an endocrine event, but also a period of microbial and metabolic remodeling [60].

These changes may influence skin aging indirectly [60,61]. Reduced estrogen availability contributes to dermal thinning, impaired barrier function, altered lipid composition, and increased inflammatory susceptibility, while gut microbiome alterations may reinforce systemic low-grade inflammation and metabolic dysfunction [60,61].

At a mechanistic level, the estrobolome includes microbial enzymes, particularly β-glucuronidases, that can deconjugate estrogens excreted into the intestine and thereby influence their reabsorption through enterohepatic circulation. This process provides a direct route through which microbial metabolism may affect systemic estrogen exposure and downstream estrogen-responsive tissues, including the skin. However, its magnitude is influenced by host hormonal status, diet, medication use, adiposity, and baseline microbiome composition [58,60].

Metabolic syndrome provides another example of endocrine–microbial interaction [61,62]. Gut dysbiosis is frequently associated with central obesity, altered glucose metabolism, dyslipidemia, hypertension, and chronic low-grade inflammation, and may contribute to intestinal inflammation, impaired metabolic signaling, and systemic inflammatory activation [61,62]. Because insulin resistance, oxidative stress, and chronic inflammation also accelerate skin aging, metabolic syndrome-related dysbiosis may indirectly contribute to cutaneous aging phenotypes [61,63,64,65,66,67]. Dietary strategies, prebiotics, probiotics, and bioactive food components have therefore been proposed to improve gut microbial balance and reduce intestinal and systemic inflammation [61,62].

#### 2.3.2. Metabolic–Endocrine Dysregulation and Inflammaging

Metabolic–endocrine dysregulation is closely linked to inflammaging, a chronic low-grade inflammatory state that contributes to age-related tissue dysfunction [63,66,67]. Hyperinsulinemia and insulin resistance are central features of metabolic dysfunction and are strongly connected to inflammatory signaling [63,67]. Obesity-associated inflammation may promote insulin resistance in metabolically active tissues such as liver, skeletal muscle, and adipose tissue, while insulin resistance may further sustain tissue inflammation, creating a self-amplifying cycle of metabolic and immune dysfunction [63,64,67].

Adipose tissue functions as an active endocrine and immune organ [64,66]. In obesity, hypertrophic adipocytes and infiltrating immune cells produce pro-inflammatory mediators, including interleukin-6 (IL-6) and tumor necrosis factor-alpha (TNF-α), which link obesity, inflammation, and metabolic disease [64,66,67]. These systemic inflammatory signals are relevant to skin aging because they may amplify oxidative stress, impair collagen homeostasis, promote AGE formation, and alter cutaneous immune responses [61,63,67].

Stress-related endocrine activation further contributes to this network [65]. Activation of the HPA axis supports acute adaptation to stress, whereas persistent stress may impair immune regulation, increase disease susceptibility, and exacerbate metabolic disorders such as diabetes, obesity, and cardiovascular disease [65,67]. In the skin, this endocrine–immune dysregulation may contribute to barrier impairment, delayed wound healing, oxidative stress, and inflammatory amplification [65,67].

Taken together, hormonal decline, metabolic dysfunction, stress axis activation, gut dysbiosis, and inflammaging form an interconnected systemic network that may influence cutaneous aging [58,60,61,62,63,64,65,66,67]. This perspective supports the rationale for microbiome-directed and bioactive interventions that target not only the skin surface, but also the broader endocrine–metabolic environment shaping skin homeostasis [52,61,62,68,69].

#### 2.3.3. Microbial Metabolites as Endocrine–Metabolic and Cutaneous Regulators

Microbial metabolites represent a key mechanistic link between microbial ecology and skin aging [10,11,12,39,40,41,46,68,69]. SCFAs, bioactive lipid metabolites, antimicrobial compounds, and other microbial-derived molecules can influence immune regulation, oxidative balance, epithelial differentiation, and barrier integrity [40,41,52,68,69]. Although these effects are best characterized in the gut, similar principles are relevant to the skin, where resident microorganisms interact with keratinocytes, sebaceous lipids, immune cells, and the stratum corneum microenvironment [10,11,12,40,41,46].

The local cutaneous mechanisms described above coexist with systemic endocrine–metabolic effects of gut- and skin-derived metabolites, including modulation of estrogen availability, gut-hormone signaling, IGF-1-related pathways, bile acid receptor activity, and immune regulation.

SCFAs, particularly acetate, propionate, and butyrate, represent an important route through which the microbiome may regulate host endocrine–metabolic signaling. In addition to their local effects on epithelial integrity and immune regulation, SCFAs can activate free fatty acid receptor 2 (FFAR2)-dependent pathways and stimulate secretion of glucagon-like peptide-1 (GLP-1) in experimental enteroendocrine models, thereby linking microbial fermentation to insulinotropic signaling and energy homeostasis [70]. Experimental studies also indicate that gut microbiota and their SCFA products can influence systemic IGF-1 signaling, providing a potential mechanistic link between microbial metabolism, anabolic signaling, and tissue remodeling [71]. In the context of skin aging, these pathways are relevant because insulin resistance, IGF-1/mTOR signaling, oxidative stress, and chronic inflammation can influence fibroblast function, ECM turnover, and barrier integrity; however, direct evidence that SCFA-induced hormonal changes modify human skin aging outcomes remains limited [23,24,25,34,35,36,37].

Other microbiome-derived metabolites may regulate systemic signaling through distinct pathways. Microbial transformation of primary bile acids alters the composition of the bile acid pool and can modify farnesoid X receptor (FXR)-dependent signaling, with downstream consequences for hepatic bile acid synthesis, glucose metabolism, lipid regulation, and inflammatory tone [72]. In addition, microbial tryptophan metabolites, including indole derivatives, can activate the aryl hydrocarbon receptor (AhR), thereby influencing epithelial differentiation, barrier function, and immune signaling. Skin-specific evidence indicates that a microbiota-derived tryptophan metabolite can attenuate cutaneous inflammation through AhR activation, although its relevance to physiological skin aging remains to be established [73].

Therefore, microbial metabolites should not be viewed solely as local cutaneous mediators. They also function as systemic endocrine–metabolic intermediates capable of influencing estrogen availability, gut-hormone signaling, IGF-1-related pathways, bile acid receptor activity, and immune regulation. This reverse regulatory arm complements the effects of hormones on microbial ecology and reinforces the bidirectional nature of the endocrine–microbiome–skin axis.

Microbial imbalance may also affect oxidative pathways [55,57]. Certain microorganisms may contribute to ROS generation or pro-oxidative enzyme activity, whereas others may produce antioxidant or photoprotective compounds [55,57]. Harel et al. showed that bacteria enriched after long sun exposure, including *Sphingomonas*, may reduce oxidative damage and protect keratinocytes against UV-induced stress [57]. Therefore, microbial ecology can influence the balance between oxidative damage and antioxidant defense, which is central to skin aging, pigmentation changes, ECM degradation, and barrier decline [55,57].

Finally, dysbiosis may contribute to ECM deterioration indirectly through inflammatory amplification [10,11,12,37,40,41]. Microbial-derived inflammatory stimuli can activate keratinocytes and immune cells, promote cytokine release, and enhance matrix metalloproteinase activity [6,10,11,12,40,41]. These processes may accelerate collagen degradation and impair dermal remodeling [6,10,11,12,37]. Thus, microbial metabolites and microbial community structure should be considered important modulators of skin homeostasis and potential targets for microbiome-directed anti-aging strategies [10,11,12,39,40,41,46,52,68,69].

**Figure 1:**
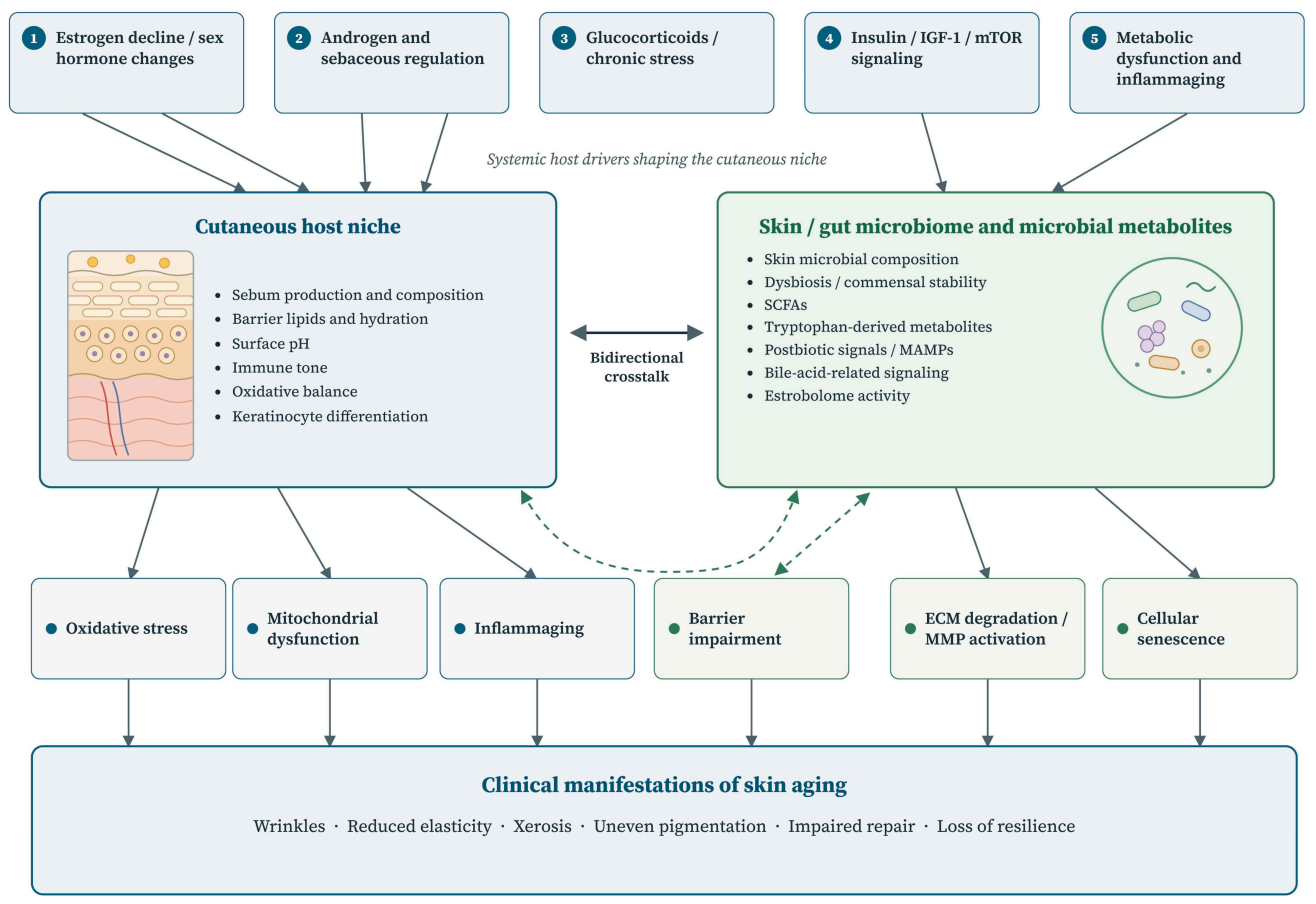
A conceptual framework of the endocrine–microbiome–skin axis in skin aging. Hormonal and metabolic drivers influence the cutaneous niche by modifying sebaceous activity, barrier lipids, hydration, surface pH, immune tone, oxidative balance, and keratinocyte differentiation. Skin- and gut microbiome-derived metabolites reciprocally influence barrier function, inflammation, oxidative stress, and endocrine–metabolic signaling. These interactions converge on oxidative stress, mitochondrial dysfunction, inflammaging, barrier impairment, extracellular matrix degradation, and cellular senescence, leading to the clinical manifestations of skin aging. The figure is an original schematic prepared using vector-based HTML graphics and exported as a high-resolution image. It synthesizes mechanisms discussed and cited in Section 1, Section 2 and Section 3 and was not adapted or reproduced from a previously published figure.

Collectively, these interactions define key intervention targets in skin aging, including ECM remodeling, oxidative stress, mitochondrial dysfunction, barrier impairment, inflammatory signaling, endocrine–metabolic dysregulation, and microbial imbalance, as summarized in Figure 1. The following section translates these interconnected pathways into molecular targets for anti-aging intervention.

## 3. Molecular Targets for Anti-Aging Intervention

From a translational perspective, the most relevant molecular targets in skin aging are located at the intersection of structural degradation, oxidative damage, barrier dysfunction, endocrine–metabolic imbalance, and microbiome dysbiosis [74]. These interconnected pathways drive wrinkles, laxity, dryness, impaired repair, and increased fragility, while also representing modifiable therapeutic targets through which targeted modulation may restore tissue homeostasis and delay cutaneous aging [74]. Rather than acting independently, oxidative stress, chronic inflammation, cellular senescence, matrix degradation, and dysbiosis reinforce one another, supporting the need for multidimensional anti-aging strategies [74].

### 3.1. ECM Remodeling Targets

ECM remodeling is one of the most accessible anti-aging targets because it directly determines dermal architecture and mechanical properties [74]. Dermal aging is characterized by an imbalance between matrix synthesis and degradation, with reduced collagen types I and III production and increased proteolytic activity [75,76]. This catabolic shift is largely driven by MMPs, which are induced by ROS and inflammatory mediators, together with attenuation of anabolic pathways such as transforming growth factor-beta (TGF-β) signaling [77,78,79]. Reduced TGF-β activity impairs fibroblast function and collagen synthesis, whereas AP-1 and NF-κB activation promotes MMP expression and accelerates ECM breakdown [79].

Anti-aging strategies targeting the ECM should therefore aim to restore the balance between matrix production and degradation. This may involve stimulation of collagen synthesis through TGF-β/Smad and PI3K/AKT signaling, suppression of MMP activity through direct or upstream antioxidant and anti-inflammatory mechanisms, and restoration of fibroblast responsiveness, which is often compromised by senescence [80,81,82]. Preserving elastin integrity is also important, as elastin fragmentation and abnormal cross-linking contribute to loss of skin elasticity [80,81,82]. Thus, ECM-directed interventions should focus not only on structural replacement, but also on fibroblast activity, controlled matrix turnover, and dermal resilience.

### 3.2. Oxidative Stress and Mitochondrial Protection

Oxidative stress and mitochondrial dysfunction are upstream drivers of skin aging and therefore represent high-impact intervention targets [5]. With aging, mitochondria show reduced oxidative phosphorylation, decreased adenosine triphosphate (ATP) and nicotinamide adenine dinucleotide (NAD+) levels, and increased ROS production [5,83]. These alterations induce molecular damage, activate pro-inflammatory signaling, and promote cellular senescence. Senescent cells acquire a senescence-associated secretory phenotype (SASP), releasing cytokines, chemokines, and proteases that amplify inflammation and matrix degradation [84]. Oxidative stress also links several hallmarks of aging, including genomic instability, telomere attrition, and inflammaging, creating a self-perpetuating cycle of tissue damage [9].

Therapeutic targeting of mitochondrial dysfunction involves enhancing endogenous antioxidant defenses, particularly through NRF2 activation, which regulates detoxifying and antioxidant enzymes [85]. Additional strategies include promoting mitochondrial biogenesis through peroxisome proliferator-activated receptor gamma coactivator 1-alpha (PGC-1α) signaling, restoring NAD+ metabolism, and improving mitophagy to maintain mitochondrial quality control [86]. By acting upstream, mitochondrial protection may reduce oxidative injury, suppress chronic inflammation, and indirectly preserve ECM integrity.

### 3.3. Barrier Integrity and Lipid Homeostasis

Barrier integrity and lipid homeostasis are critical targets because the epidermal barrier maintains hydration, protects against environmental insults, and regulates immune responses [76]. Aging is associated with quantitative and qualitative changes in epidermal lipids, including ceramides, cholesterol, and FFAs, contributing to increased TEWL and skin dryness [87]. Reduced expression of structural proteins such as filaggrin further impairs keratinocyte differentiation and stratum corneum formation [8]. Sebaceous gland aging, characterized by progressive atrophy, reduced sebum production, and altered lipid composition, contributes to xerosis, roughness, and decreased skin resilience [87,88]. These alterations weaken the physical barrier and facilitate the penetration of irritants and microbial products, thereby promoting inflammation and accelerating aging processes [87].

Restoring barrier function requires more than hydration; it involves re-establishing the biochemical and structural integrity of the epidermis [76,87]. Relevant approaches include normalization of ceramide balance, enhancement in lipid synthesis pathways, modulation of sebaceous gland activity, and support of keratinocyte differentiation [76,87]. By reinforcing barrier integrity, these strategies may reduce inflammation, improve hydration, and create a more stable microenvironment resistant to intrinsic and extrinsic aging factors [76,87].

### 3.4. Microbiome Modulation

Microbiome modulation is a relevant anti-aging strategy because resident microbial communities contribute to barrier integrity, immune regulation, antimicrobial defense, and metabolite signaling [12]. Aging-associated dysbiosis may reduce commensal functions, increase inflammation-associated taxa, and disrupt SCFA and antimicrobial-peptide-related support for surface acidity, barrier homeostasis, and oxidative balance [12,89,90,91,92]. Accordingly, probiotics, prebiotics, and postbiotics aim to restore ecological balance rather than indiscriminately eradicate microorganisms, although their clinical effects remain intervention-, formulation-, and host-specific [91,92,93,94].

Taken together, ECM remodeling, mitochondrial function, barrier integrity, and microbiome balance represent interconnected targets that define the aging phenotype of the skin. Targeting these interconnected pathways may offer a more comprehensive anti-aging strategy by addressing both visible structural alterations and the underlying biological processes that drive skin aging.

### 3.5. Endocrine and Metabolic Targets in Skin Aging

Endocrine and metabolic pathways are actionable anti-aging targets because they regulate tissue repair, ECM turnover, oxidative stress, immune balance, energy metabolism, and stress adaptation [95]. Modulation of estrogen receptor activity, IGF-1 signaling, insulin sensitivity, cortisol regulation, and hormone–immune interactions may therefore provide complementary strategies for preserving skin structure and function during aging [95].

#### 3.5.1. Estrogen Receptor Activation Pathways

Estrogen receptor signaling is one of the most relevant endocrine targets in skin aging, particularly because estrogen levels decline with age in both women and men [96,97,98,99]. Among estrogenic ligands, 17β-estradiol (E2) is especially important because it supports tissues with high remodeling capacity, including the skin, bone, and brain [100,101,102]. Estrogen deficiency is associated with osteoporosis, cognitive decline, impaired wound healing, and experimental models of aging-associated tissue dysfunction [103,104,105].

In the skin, estrogen receptor activation supports collagen and elastin production, hydration, vascularization, hair growth, and ECM homeostasis [42]. After menopause, declining estrogen levels contribute to thinner, drier, and less elastic skin, reduced collagen content, decreased mechanical strength, and delayed wound healing [16,106]. Estrogen signaling may also improve wound healing and inflammatory control by regulating cytokine production, oxidative stress, cell proliferation, angiogenesis, and ECM formation [101,107]. Although estrogen replacement has been associated with improved skin quality and accelerated healing, its effects on scar formation remain complex and incompletely defined [16].

At the cellular level, estrogen signaling may counter biological aging by reducing oxidative stress and apoptosis, supporting mitochondrial function, modulating epigenetic regulation, preserving genome stability, and contributing to telomere maintenance [108,109,110,111,112,113]. Estrogen metabolites, including catechol and methoxy estrogens, may also contribute to vascular regulation and angiogenesis, although their skin-specific role remains insufficiently characterized [114].

Estrogen acts through classical genomic pathways involving estrogen receptor alpha (ERα) and estrogen receptor beta (ERβ), as well as non-genomic pathways mediated by membrane-associated receptors and G protein-coupled estrogen receptor (GPER) [115,116,117,118,119]. Fibroblasts, keratinocytes, immune cells, stem cells, and endothelial cells express estrogen receptors and may respond directly to estrogenic modulation [107]. Experimental models show that estrogen signaling is activated during wound healing, reduced by estrogen deprivation, and restored by E2 treatment [116]. ERβ appears particularly important for keratinocyte migration, wound closure, and inflammation control, whereas ERα mainly contributes to anti-inflammatory and remodeling processes [117,118]. Estrogen may also act through ERK/AKT signaling, IGF-1 receptor activation, and GPER, supporting keratinocyte proliferation and repair [120]. Overall, estrogen effects in skin are mediated by overlapping receptor-dependent and non-canonical pathways, although their precise roles in homeostasis, aging, and repair remain incompletely defined [95].

#### 3.5.2. IGF-1 Signaling and mTOR Balance

IGF-1 signaling is an important target for modulating skin aging because it regulates cellular proliferation, differentiation, survival, and tissue repair [121]. IGF-1 is a major mediator of growth hormone activity and acts through the IGF-1 receptor to activate downstream PI3K/AKT and MAPK signaling cascades [121]. The IGF-1 system supports fetal development, postnatal growth, adult tissue homeostasis, cardiovascular and nervous system function, muscle regeneration, and skeletal metabolism [122].

From an anti-aging perspective, IGF-1 should be viewed as a context-dependent target rather than a pathway that should be uniformly stimulated or suppressed [122,123]. Reduced IGF-1 signaling may contribute to impaired tissue regeneration, whereas excessive IGF-1/mTOR activation may promote uncontrolled proliferation, cancer risk, and age-associated metabolic stress through insulin receptor substrate (IRS)-1, IRS-2, and PI3K/AKT/mTOR signaling [122,123]. Therefore, therapeutic modulation of IGF-1 signaling remains relevant to oncology, endocrinology, longevity research, and skin repair biology [122,123].

#### 3.5.3. Insulin Sensitivity, Hyperglycemia, and Metabolic Intervention

Insulin sensitivity is relevant to skin aging because insulin resistance promotes systemic inflammation, oxidative stress, and metabolic dysfunction [122]. Insulin resistance, characteristic of type 2 diabetes mellitus and obesity, involves impaired cellular responsiveness to insulin, reduced glucose uptake, and dysregulated metabolism [122]. At the molecular level, insulin signaling depends on activation of the insulin receptor and downstream IRS-1/PI3K/AKT pathways, which regulate glucose uptake through glucose transporter type 4 (GLUT4) translocation [122]. Chronic inflammation, lipid accumulation, and oxidative stress interfere with these pathways and worsen insulin resistance [122].

Improving insulin sensitivity through lifestyle strategies, including diet and physical activity, or pharmacological agents such as metformin and thiazolidinediones may improve glycemic control, reduce cardiovascular risk, and lower systemic inflammation [122]. These effects are relevant to age-related skin deterioration because chronic hyperglycemia, oxidative stress, and metabolic inflammation promote collagen damage, impaired repair, and accelerated tissue aging [122].

#### 3.5.4. Cortisol Regulation and Cutaneous Stress Signaling

Cortisol regulation represents an anti-aging target because chronic stress signaling affects metabolism, immunity, circadian rhythm, barrier integrity, and inflammatory balance [123]. Cortisol, the primary glucocorticoid hormone, is secreted under HPA axis control and regulates glucose metabolism, immune function, and circadian rhythm [26]. Although acute cortisol release is adaptive, chronic elevation may contribute to insulin resistance, central adiposity, immune suppression, and neuropsychiatric disorders [124].

In the skin, stress responses are mediated through both the central HPA axis and a peripheral HPA-like system [26]. Keratinocytes, melanocytes, fibroblasts, and sebocytes can produce CRH, ACTH, and related receptors in response to stressors such as UV radiation and inflammation [26]. Through glucocorticoid receptor signaling, cortisol influences inflammatory, immune, and metabolic pathways, while CRH and ACTH modulate keratinocyte proliferation, differentiation, melanogenesis, sebocyte activity, mast cell activation, and inflammation [26]. Chronic stress and dysregulated cortisol signaling may therefore impair skin homeostasis, accelerate aging processes, and exacerbate inflammatory skin conditions [26].

#### 3.5.5. Hormone–Immune Interactions

Hormone–immune interactions represent a central target for controlling inflammaging and age-associated tissue dysfunction [125,126]. The endocrine and immune systems are connected through bidirectional signaling networks that maintain immune homeostasis and regulate inflammatory responses [125,126]. Glucocorticoids generally exert anti-inflammatory and immunosuppressive effects by modulating glucocorticoid receptor signaling and inhibiting NF-κB- and AP-1–dependent gene expression [125]. Estrogens exert context-dependent immunomodulatory effects that may enhance or suppress immune activity depending on receptor expression, hormone concentration, and tissue microenvironment [126].

Conversely, immune-derived cytokines such as IL-6 and TNF-α can activate the HPA axis, reinforcing endocrine stress responses and creating a feedback loop between immune activation and hormonal regulation [125]. This bidirectional crosstalk is relevant in chronic inflammatory disorders, autoimmune diseases, and metabolic dysregulation, where persistent stress signaling contributes to immune imbalance and disease progression [125].

Overall, endocrine and metabolic modulation should be considered a multidimensional anti-aging strategy rather than a single-pathway intervention [95]. Estrogen receptor activation, IGF-1 pathway modulation, improved insulin sensitivity, cortisol regulation, and control of hormone–immune crosstalk may converge on improved ECM maintenance, reduced oxidative stress, enhanced tissue repair, better immune regulation, and preservation of skin homeostasis [95].

The molecular targets described above provide the mechanistic rationale for selecting anti-aging bioactive compounds. Because individual compounds may act simultaneously on oxidative stress, ECM remodeling, barrier function, inflammation, endocrine signaling, or microbial homeostasis, the next section evaluates microbiome-directed and multifunctional bioactives according to their principal mechanisms, level of evidence, and translational limitations.

## 4. Bioactive Compounds with Multidimensional Activity

Bioactive compounds used in anti-aging strategies increasingly extend beyond single-target cosmetic effects. Many may simultaneously influence oxidative stress, inflammation, ECM remodeling, mitochondrial dysfunction, barrier integrity, endocrine–metabolic signaling, and microbiome composition. This multidimensional activity is particularly relevant in skin aging, where structural degradation, inflammaging, dysbiosis, and impaired repair mechanisms reinforce one another. Accordingly, the following sections examine the principal bioactive categories according to their dominant mechanisms, target pathways, and translational relevance.

To clarify the level of evidence supporting each intervention, the studies discussed in this section are classified according to the highest available study modality: controlled human clinical evidence, uncontrolled or observational human evidence, in vivo preclinical evidence, ex vivo/in vitro evidence, or formulation/mechanistic evidence. Human studies are discussed separately from experimental data whenever possible. Therefore, mechanistic, cell-based, animal, and delivery-platform findings should be interpreted as biological plausibility or proof-of-concept rather than confirmation of clinical anti-aging efficacy.

### 4.1. Microbiome-Targeted Interventions: Definitions, Mechanisms, Evidence, and Dermocosmetic Translation

Microbiome-targeted dermo-nutraceutical and dermocosmetic strategies include probiotics, prebiotics, postbiotics, and synbiotics. In accordance with International Scientific Association for Probiotics and Prebiotics definitions, probiotics are live microorganisms that confer a health benefit when administered in adequate amounts; prebiotics are substrates selectively utilized by host microorganisms that confer a health benefit; postbiotics are preparations of inanimate microorganisms and/or their components that confer a health benefit; and synbiotics combine live microorganisms with selectively utilized substrates [127,128,129,130,131].

These terms require careful use in dermatological and dermocosmetic applications. Products containing lysates, ferment filtrates, or non-viable microbial components should not be labeled “probiotic” unless they contain viable, adequately characterized microorganisms at an effective dose and demonstrate a host benefit. Likewise, a topical ingredient should be considered a prebiotic only when selective utilization within the cutaneous microbiome and a measurable host benefit are demonstrated. Ferment-derived ingredients, bacterial lysates, and isolated microbial metabolites may be promising microbial-derived actives, but should be classified as postbiotics only when they meet the accepted compositional and functional criteria [127,128,129,130,131,132].

This distinction is relevant because biological activity depends on strain identity, viability, dose, formulation, preservative compatibility, stability, route of administration, and host context. The technical difficulty of maintaining viable microorganisms in topical formulations has therefore increased interest in postbiotic and microbial-derived platforms [128,132].

#### 4.1.1. Route-Specific Mechanisms Relevant to Skin Aging

Oral and topical microbiome-targeted interventions may influence skin homeostasis through partially distinct mechanisms. Oral probiotic, prebiotic, and synbiotic strategies primarily act through the gut–skin axis, where changes in microbial composition and metabolite production may influence systemic inflammation, SCFA availability, oxidative stress, and epithelial barrier regulation. Selected *Lactobacillus* and *Bifidobacterium* strains may increase circulating SCFAs, including acetate, propionate, and butyrate, which can support keratinocyte metabolism, epithelial differentiation, barrier integrity, and inflammatory regulation [69].

Topical interventions act predominantly at the skin surface, stratum corneum, and follicular openings. Their proposed effects include modulation of microbial competition, support of barrier-related proteins, reduction in inflammatory signaling, and protection against oxidative or environmental stress. For example, *Bifida ferment* lysate increased the expression of filaggrin, loricrin, involucrin, transglutaminase-1, aquaporin-3, cathelicidin, and β-defensin-2 in keratinocyte models [132]. However, microbial metabolites, exopolysaccharides, lysates, and strain-specific components should not be generalized across probiotic taxa because their biological effects remain dependent on the source microorganism, processing method, dose, formulation, and delivery route [133,134].

#### 4.1.2. Evidence from Human, Preclinical, and In Vitro Studies

Controlled human evidence. In a randomized, double-blind, placebo-controlled trial in women with photoaged skin, *Lactobacillus plantarum* HY7714 reduced wrinkle depth, improved skin elasticity, and reduced TEWL [135]. Two additional randomized, double-blind, placebo-controlled studies reported that oral *Bifidobacterium breve* M-16V prevented worsening of total VISIA score and improved brown spot and pore scores, whereas heat-treated *Latilactobacillus sakei* KABP-065 improved cheek skin elasticity over 8 weeks, particularly in a prespecified subgroup of women in their 40s [136,137].

Preclinical evidence. *Bifidobacterium breve* Yakult preserved stratum corneum hydration and reduced UV -induced oxidative stress in a hairless mouse model, whereas *Bifidobacterium longum* combined with galacto-oligosaccharides attenuated ultraviolet B (UVB)-induced photoaging in experimental models through SCFA-related and antioxidant pathways [138,139].

In vitro evidence. *Bifida ferment* lysate increased the expression of several barrier-related and antimicrobial proteins in keratinocyte models [132]. These data support biological plausibility but should not be interpreted as evidence of clinical anti-aging efficacy.

Controlled human evidence. A randomized, double-blind, placebo-controlled study of 2% LactoSporin cream reported reductions in visible wrinkles and fine lines, although hydration, transepidermal water loss, and elasticity outcomes were not uniformly superior to placebo [140]. LactoSporin is an extracellular microbial-derived ingredient from *Bacillus coagulans* rather than a live probiotic formulation; its classification should therefore be interpreted according to its final compositional characteristics and the accepted definition of postbiotics [140].

Synbiotic and prebiotic strategies may complement probiotic or postbiotic approaches by supporting beneficial microbial metabolism. An oral synbiotic containing galacto-oligosaccharides plus *B. breve* improved facial skin moisture parameters and modified gut microbial composition in healthy women [12,141,142]. The same combination also reduced serum levels of phenolic gut-derived metabolites relevant to skin aging [141,142]. Prebiotic polyphenols may further influence microbial composition and metabolite production, including pathways involving *Akkermansia muciniphila* and *Faecalibacterium prausnitzii* [143]. Nevertheless, direct evidence that topical prebiotic formulations improve established clinical skin aging endpoints remains limited.

#### 4.1.3. Dermocosmetic Platforms and Translational Considerations

The commercial landscape includes oral strain-specific supplements, fermented milk products, topical lysates, ferment-derived ingredients, microbial metabolites, and formulations containing putative prebiotic substrates such as inulin, fructo-oligosaccharides, galacto-oligosaccharides, or alpha-glucan oligosaccharides. However, commercial availability should not be equated with scientific validation. Many marketed “probiotic skincare” products contain non-viable lysates or fermentation-derived ingredients rather than live probiotics, and product labels often do not report strain identity, viable count at the end of shelf life, manufacturing process, active fraction, or microbiome-related clinical outcomes. Representative intervention platforms and their evidence boundaries are summarized in Table 1.

Despite increasing interest, clinical translation remains limited by methodological heterogeneity, small sample sizes, limited study duration, insufficient data in adults aged 60 years or older, and inconsistent characterization of intervention composition [144].

Most available human studies are short-term and use heterogeneous cosmetic, biophysical, and microbiome-related endpoints; therefore, they cannot yet establish the durability of benefit, clinically meaningful modification of skin aging trajectories, or long-term effects on the cutaneous and gut microbial ecosystem. Larger, independently replicated trials with standardized formulations, longer follow-up, and harmonized efficacy and safety outcomes are needed.

Strain specificity is particularly important because clinical effects cannot be extrapolated across strains within the same species. For live probiotics, future studies should report strain identity, colony-forming-unit dose at the end of shelf life, viability, storage conditions, administration route, and formulation matrix. For postbiotic or microbial-derived preparations, the producing strain, manufacturing process, active fraction, compositional fingerprint, and batch-to-batch reproducibility should be reported [127,128,129,130,144].

Future work should also distinguish between evidence of microbiome modulation, barrier support, and clinically meaningful anti-aging benefit. This distinction is essential for avoiding overgeneralized cosmetic claims and for developing evidence-based microbiome-directed interventions.

Although microbiome-targeted interventions primarily aim to restore microbial and barrier homeostasis, other bioactive compounds may influence the endocrine, oxidative, and ECM pathways that interact with the microbiome. Phytoestrogens and polyphenols are particularly relevant in this context because they combine antioxidant and anti-inflammatory activity with endocrine-responsive and microbiome-related mechanisms.

### 4.2. Phytoestrogens and Polyphenols

#### 4.2.1. Phytoestrogens: Genistein and Daidzein

Phytoestrogens are plant-derived compounds structurally and functionally similar to endogenous estrogens, among which the soy isoflavones genistein and daidzein are the most extensively studied [145]. Their relevance in skin aging is linked to their ability to interact with estrogen receptors and modulate endocrine function, dermal homeostasis, and age-related physiological changes [145].

Genistein and daidzein act primarily through estrogen receptor binding, with preferential affinity for ERβ over ERα, although their affinity is weaker than that of endogenous E2 [145,146]. This ERβ preference is relevant because ERβ is expressed in several non-reproductive tissues, including skin, bone, and the cardiovascular system [145,146]. Genistein has higher binding affinity than daidzein and behaves as a partial agonist, allowing for tissue-specific estrogenic or anti-estrogenic effects depending on endogenous hormonal status [145]. Beyond estrogen receptor binding, phytoestrogens may also influence tyrosine kinase activity and growth factor signaling pathways [145,147].

In dermatology, the most relevant anti-aging mechanism of phytoestrogens is their potential to support collagen homeostasis. Estrogen promotes dermal collagen synthesis, and its decline during aging contributes to reduced elasticity and wrinkle formation [147]. Genistein may stimulate fibroblast activity and collagen production through TGF-β signaling and may inhibit MMPs involved in collagen degradation [148]. Daidzein appears less potent than genistein but may also support ECM maintenance through antioxidant, anti-inflammatory, and gene-regulatory mechanisms [149]. These effects suggest that phytoestrogens may help preserve dermal structure, particularly in estrogen-deficient individuals [145].

Limited controlled human evidence is available, and findings remain heterogeneous. In postmenopausal women, soy isoflavone supplementation has been associated with improvements in skin elasticity, hydration, and wrinkle depth [150]. Randomized controlled trials have reported that genistein supplementation may increase collagen content and improve skin thickness, supporting its potential as a non-hormonal alternative to estrogen therapy [151]. However, outcomes depend on dose, treatment duration, baseline hormonal status, and individual metabolism, including the ability to convert daidzein into equol [152]. Although phytoestrogens are generally considered safe, the long-term effects of high-dose supplementation remain insufficiently defined and require caution, especially in hormone-sensitive populations [152].

#### 4.2.2. Polyphenols and Microbiome Modulation

Beyond phytoestrogens, dietary polyphenols such as resveratrol, curcumin, epigallocatechin gallate, anthocyanins, and ellagic acid exert anti-aging effects through direct antioxidant/anti-inflammatory activity and indirect modulation of the gut microbiome [143]. Because many polyphenols have low intrinsic bioavailability, they reach the colon and are biotransformed by resident bacteria into metabolites such as urolithins, equol, valerolactones, and phenolic acids, which may display greater biological activity than the parent compounds while also promoting beneficial microbial taxa [143].

A recent 8-week randomized, double-blind, placebo-controlled trial in women aged ≥40 years showed that combined oral trans-resveratrol at 75 mg/day and topical trans-resveratrol at 1.5% significantly reduced facial wrinkle scores compared with placebo, with serum trans-resveratrol conjugates confirming systemic absorption [153]. This trial supports the potential integration of polyphenol-based nutricosmetics with topical formulations, although additional independent studies are needed to confirm the clinical relevance of dual-route delivery [153].

Whereas phytoestrogens and polyphenols mainly act through endocrine-responsive, antioxidant, and microbiome-modulating pathways, bioactive peptides provide a more direct strategy for influencing fibroblast activity, ECM synthesis, and tissue repair. This distinction is relevant because preservation of dermal structure requires both upstream control of inflammatory and oxidative stressors and direct support of matrix homeostasis.

### 4.3. Bioactive Peptides and Growth Factor Modulators

Bioactive peptides used in cosmeceuticals are commonly classified as signal or matrikine peptides, carrier peptides, neurotransmitter-inhibiting peptides, and enzyme inhibitor peptides. Matrikines are bioactive fragments generated by proteolytic cleavage of ECM proteins, including collagens, elastin, laminin, and fibronectin, and can modulate cell adhesion, migration, proliferation, angiogenesis, and protein expression in the skin [154]. This concept provides the rationale for synthetic anti-aging peptides designed to mimic endogenous regenerative signals [154].

#### 4.3.1. Glycyl-L-Histidyl-L-Lysine Copper Complex (GHK-Cu): Copper Tripeptide-1

The naturally occurring tripeptide glycyl-L-histidyl-L-lysine, when complexed with copper (II), is one of the most extensively characterized cosmeceutical peptides [155]. Plasma GHK-Cu concentrations decline by approximately 60% between ages 20 and 60, paralleling reduced regenerative capacity [155]. GHK-Cu modulates the expression of more than 4000 genes, upregulating pathways involved in DNA repair, antioxidant defense, ECM synthesis, stem cell function, and tissue regeneration, while downregulating inflammatory mediators [155]. Mechanistic, in vitro, and product-specific human evidence supports effects on fibroblast proliferation and MMP modulation; small topical-formulation studies have also reported improvements in wrinkle depth, density, and barrier-related outcomes [155]. However, large independent randomized controlled trials are lacking.

#### 4.3.2. Procollagen-Derived Signal Peptides: KTTKS and Palmitoylation Strategies

Palmitoyl-pentapeptide-4, also known as Pal-KTTKS or Matrixyl^®^, is derived from the C-terminal propeptide of type I procollagen and stimulates collagen I, collagen III, and fibronectin synthesis in dermal fibroblasts [156]. In a 12-week double-blind, placebo-controlled, split-face randomized trial, topical 3 ppm Pal-KTTKS in moisturizer significantly reduced fine lines and wrinkles compared with vehicle [156]. The synthetic tetrapeptide GEKG, palmitoyl-glycyl-glutamyl-lysyl-glycyl, has also been shown to enhance ECM formation in vitro and improve clinical skin parameters [157]. A broader review of growth factor, cytokine, and matrikine cosmeceutical formulations summarizes the translational evidence supporting this peptide class [158].

#### 4.3.3. Oral Collagen Peptides: Critical Assessment of Clinical Evidence

Controlled human studies have evaluated hydrolyzed collagen peptides, typically administered at 2.5–10 g/day for 8–12 weeks, for hydration, elasticity, and wrinkle-related outcomes [159]. VERISOL^®^ supplementation at 2.5 g/day for 8 weeks reduced eye-area wrinkle volume and increased dermal procollagen I and elastin synthesis in a double-blind, placebo-controlled trial of women aged 45–65 years [159]. A randomized, double-blind, placebo-controlled trial of low-molecular-weight collagen peptide at 1650 mg/day for 8 weeks reported improvements in wrinkles, hydration, elasticity, and dermal density [160]. However, a recent meta-analysis of 23 randomized controlled trials including 1474 participants found that significant benefits were largely confined to industry-funded studies, while high-quality independently funded trials showed no significant effect [161]. A subsequent commentary further emphasized the methodological implications of commercial sponsorship in collagen peptide research [162].

### 4.4. Antioxidants and Mitochondrial Protectors

Antioxidants and mitochondrial protectors target oxidative damage, mitochondrial dysfunction, lipid peroxidation, photoaging-associated inflammation, and impaired collagen homeostasis [163,164,165,166,167,168,169,170,171,172]. Their translational relevance depends not only on antioxidant capacity, but also on chemical stability, skin penetration, bioavailability, delivery route, and formulation compatibility [163,164,165,166,167,168,169,170,171,172]. The main compounds, mechanisms, evidence level, and translational limitations are summarized in Table 2.

### 4.5. Adaptogens and Metabolic Modulators

Adaptogens and endocrine–metabolic modulators represent a heterogeneous group of bioactive compounds that may influence skin aging indirectly through stress axis regulation, metabolic homeostasis, immune modulation, oxidative stress control, and circadian signaling [173,174]. Their relevance lies in their capacity to modulate upstream drivers of barrier dysfunction, inflammaging, insulin resistance, mitochondrial impairment, and impaired tissue repair [173,174]. These compounds mainly act through neuroendocrine systems, particularly the HPA axis, while also interacting with metabolic and immune signaling networks [173,174].

#### 4.5.1. HPA Axis, Circadian, and Endocrine–Immune Modulation

The HPA axis is a central regulator of the stress response, and chronic activation is associated with insulin resistance, immune dysregulation, circadian disruption, and accelerated biological aging [175]. Botanical adaptogens such as *Withania somnifera*, *Rhodiola rosea*, and *Panax ginseng* may influence HPA axis activity and improve stress adaptation, with clinical evidence showing that *Withania somnifera* can reduce circulating cortisol and improve stress-related outcomes [173,175]. At the molecular level, adaptogens may regulate stress response mediators, including heat shock protein 70 (Hsp70), c-Jun N-terminal kinase 1 (JNK1), forkhead box O (FoxO) transcription factors, cortisol, and nitric oxide, thereby supporting resilience under fatigue and potentially mitigating stress-induced neuroendocrine and immune dysfunction [175]. These mechanisms are relevant because chronic stress contributes to oxidative damage, inflammatory amplification, impaired barrier function, and delayed repair in the skin [175].

Melatonin is a multifunctional hormone involved in circadian rhythm regulation, metabolic signaling, immune modulation, mitochondrial function, and antioxidant defense [176,177]. Through MT1 and MT2 receptors, melatonin regulates glucose metabolism and insulin secretion, while reduced melatonin synthesis or altered receptor activity has been associated with sleep disruption, metabolic dysfunction, immune imbalance, and oxidative stress [176,177]. In dermatology, melatonin is relevant because circadian disruption, oxidative stress, mitochondrial dysfunction, and impaired DNA repair contribute to photoaging and barrier decline [176,177]. These findings support further investigation of melatonin for photoprotection, oxidative stress reduction, and maintenance of skin barrier integrity, although evidence for broader anti-aging effects remains heterogeneous [176,177].

Vitamin D functions as a secosteroid hormone involved in calcium metabolism, immune regulation, endocrine signaling, and skin homeostasis [178]. Its active form, calcitriol, binds to the vitamin D receptor, which is widely expressed in immune and endocrine tissues [178]. Vitamin D modulates cytokine production, promotes immune tolerance, enhances innate immune responses, supports regulatory T-cell activity, and suppresses excessive Th1, Th17, and B-cell activation [179]. Deficiency has been associated with impaired immune balance, autoimmune disease, metabolic syndrome, altered stress responses, and increased inflammatory susceptibility [179]. The existing literature also suggests that vitamin D may modulate HPA axis activity and cortisol dynamics, positioning it as a mediator at the endocrine–immune interface [178,179]. In the skin aging context, these effects are relevant because immune dysregulation, chronic inflammation, oxidative stress, and impaired barrier defense are central components of cutaneous aging [178,179].

#### 4.5.2. Insulin-Sensitizing and AMP-Activated Protein Kinase (AMPK)-Activating Compounds

Insulin-sensitizing compounds are relevant to skin aging because insulin resistance, hyperglycemia, oxidative stress, and chronic inflammation contribute to collagen glycation, impaired repair, and accelerated tissue dysfunction [180]. This category includes conventional pharmacological agents such as biguanides and thiazolidinediones, as well as naturally derived molecules including berberine and several polyphenols [180]. These substances may improve metabolic function through AMPK activation, attenuation of oxidative stress, and regulation of inflammatory pathways [180]. Natural compounds, particularly resveratrol, curcumin, flavonoids, and berberine, are increasingly explored as complementary metabolic modulators, with berberine showing effectiveness in improving glycemic control and lipid metabolism in individuals with type 2 diabetes [180].

In skin aging, improved metabolic control may reduce systemic inflammatory burden, limit glycation-related collagen stiffening, and attenuate oxidative damage [180]. Adaptogenic compounds may also indirectly support insulin sensitivity by mitigating chronic cortisol elevation, a factor known to disrupt insulin signaling [180]. This interaction supports the potential value of combining metabolic, stress axis, and antioxidant strategies, although direct evidence linking systemic metabolic improvements to clinically meaningful cutaneous anti-aging outcomes remains limited [180].

#### 4.5.3. Safety, Dosing, and Translational Limitations

Despite their therapeutic potential, adaptogens and metabolic modulators can influence endocrine function through interactions with hormone receptors, nuclear receptor pathways, and intracellular signaling cascades [181]. Certain compounds may display hormone-like activity or interfere with endocrine signaling, resulting in context-dependent effects on hormonal balance [181]. Variability in exposure, formulation, standardization, study design, and confounding factors makes it difficult to establish clear cause–effect relationships [181]. Therefore, receptor-level interactions should be carefully assessed, particularly for compounds with phytohormonal properties or those used over extended periods [181]. Although many of these agents are generally considered safe, their effects may depend on dosage, duration, formulation, baseline endocrine status, comorbidities, concomitant medication, and individual susceptibility [181].

Dosing varies according to compound, formulation, clinical indication, and population [181]. Common clinical ranges include 300–600 mg/day of standardized *Withania somnifera* extract for 8–12 weeks, with higher escalating doses of 750–1250 mg/day also reported as tolerated [182]. Melatonin is commonly used systemically at 0.5–5 mg/day for sleep disorders, while topical formulations have been investigated for photoprotection and selected skin conditions [183]. Vitamin D is often administered at 1000–4000 IU/day for maintenance of sufficiency, although higher doses may be used in selected therapeutic contexts under clinical supervision [184]. Berberine is commonly administered at approximately 500 mg two to three times daily in metabolic studies [185]. Individualized dosing should consider baseline endocrine status, circadian rhythm, metabolic condition, clinical indication, and long-term safety [181].

Clinical endocrinology research supports the role of adaptogens and metabolic modulators in improving stress resilience, metabolic function, and immune regulation [173,174,175]. Melatonin and vitamin D have consistent roles in regulating oxidative stress, immune responses, and endocrine signaling, with emerging relevance for systemic and dermatological applications [176,177,178,179]. However, variability in study design, dosing protocols, extract standardization, population characteristics, and outcome measures limits the generalizability of current findings [176,177,178,179]. For skin aging, the strongest rationale for this class lies in modulation of upstream systemic drivers, including chronic stress, circadian disruption, insulin resistance, oxidative stress, and endocrine–immune imbalance [173,174,175,176,177,178,179,180]. Further large-scale, long-term studies are required to determine which compounds have direct, clinically meaningful effects on cutaneous aging endpoints [176,177,178,179].

The principal bioactive classes discussed in Section 4, together with their dominant biological targets and relative evidence maturity, are summarized in Table 3.

Table 3 shows that the bioactive categories discussed in this review differ substantially in evidence maturity. Selected probiotics, collagen-derived compounds, peptides, antioxidants, and phytoestrogens are supported by some controlled human evidence, although findings remain compound-, strain-, formulation-, and population-specific. In contrast, several prebiotic, synbiotic, adaptogenic, metabolic-modulating, and nanodelivery-related approaches remain primarily supported by mechanistic, preclinical, ex vivo, or formulation-based evidence. Therefore, comparative interpretation should consider not only biological plausibility, but also study design, endpoint quality, replication, and clinical relevance.

Despite their mechanistic potential, many bioactive compounds are limited by instability, poor solubility, oxidation, low skin retention, or insufficient access to the intended cutaneous compartment. These formulation-related constraints provide the rationale for the advanced delivery systems discussed in the following section.

## 5. Advanced Delivery Systems for Precision Anti-Aging Targeting

Advanced delivery systems are increasingly relevant in anti-aging research because many bioactive compounds, including polyphenols, peptides, antioxidants, retinoids, probiotics, and postbiotics, show limited stability, poor skin penetration, rapid degradation, or insufficient localization within the target cutaneous compartment. Nanocarrier-based systems are designed to improve physicochemical stability, enhance cutaneous deposition, prolong release, and increase site-specific activity. However, most evidence remains formulation-based, ex vivo, preclinical, or product-specific, and clinical anti-aging efficacy should therefore be interpreted cautiously.

### 5.1. Liposomes and Phospholipid Vesicular Systems

Liposomes are spherical vesicles composed of one or more phospholipid bilayers surrounding an aqueous core, allowing for encapsulation of hydrophilic compounds within the core and lipophilic compounds within the bilayer [186]. Their amphipathic structure supports their use in cosmeceutical delivery, particularly for unstable or poorly soluble anti-aging actives such as polyphenols, peptides, antioxidants, and retinoids [186]. At the skin interface, liposomes may interact with stratum corneum lipids, protect actives from oxidation or pH-mediated degradation, and improve cutaneous deposition, although true dermal penetration through intact skin remains limited [187].

Liposomal encapsulation can enhance the stability of labile compounds such as L-ascorbic acid, resveratrol, epigallocatechin gallate (EGCG), ergothioneine, retinoids, vitamin D3, and ellagic acid [188,189,190,191]. Representative studies have shown improved colloidal stability, encapsulation efficiency, skin retention, and experimental penetration compared with free actives [188,189,190,191]. However, conventional liposomes accumulate mainly in the upper stratum corneum and rarely reach the viable epidermis or dermis in significant quantities [192].

This limitation has driven the development of ultra-deformable vesicles, including transfersomes, ethosomes, and transethosomes [192,193]. Transfersomes incorporate edge activators such as sodium deoxycholate, sodium cholate, or polysorbate 80 to increase vesicle elasticity and facilitate passage through narrow intercellular channels [193]. EGCG–hyaluronic acid nano-transfersomes enhanced ex vivo skin permeation and more effectively suppressed UVB-induced ROS, MMP-2, and MMP-9 in keratinocytes compared with free actives [194]. Ethosomes use ethanol to fluidize vesicular and stratum corneum lipids, whereas transethosomes combine ethanol and edge activators to further improve deformability and penetration, although clinical anti-aging evidence remains limited [187,191,192].

Vesicular systems may also support transfollicular targeting because liposomes and related carriers can accumulate within pilosebaceous structures relevant to epidermal renewal, sebaceous biology, and microbiome modulation [195]. Multi-cargo liposomal systems carrying peptides, antioxidants, and retinoid-like actives are mechanistically attractive, but current evidence supports formulation feasibility and enhanced penetration more strongly than definitive clinical anti-aging efficacy [187,196,197].

From a safety perspective, phosphatidylcholine, cholesterol, and related lipids are generally biocompatible and biodegradable, but vesicle composition, charge, particle size, excipient load, and release behavior require formulation-specific assessment [186,198,199,200]. A liposome-encapsulated antioxidant complex did not induce cytotoxicity or metabolic stress in UV-irradiated human skin explants and normalized IL-6, IL-8, and MMP-9 toward basal levels [198,199]. Cationic liposomes may carry greater toxicological risk, while high ethanol content in ethosomes may be irritating in rosacea, atopic dermatitis, or impaired barrier function [193,200]. EU cosmetic safety assessment requires characterization of particle size, encapsulation efficiency, zeta potential, membrane composition, and release behavior [186].

### 5.2. Solid Lipid Nanoparticles and Nanostructured Lipid Carriers

Solid lipid nanoparticles (SLNs) consist of a crystalline solid lipid matrix stabilized by surfactants, whereas nanostructured lipid carriers (NLCs) combine solid and liquid lipids to create a less ordered matrix with higher loading capacity and reduced drug expulsion during storage [201,202]. NLCs can be designed as imperfect, multiple, or amorphous systems, allowing release kinetics to be adapted to the active compound and intended cutaneous target [202].

The solid or semi-solid lipid matrix protects oxidation-sensitive anti-aging compounds from oxygen, light, and enzymatic degradation [201,202,203,204]. Coenzyme Q10 (CoQ10)-loaded NLCs showed encapsulation efficiency above 80% and better physicochemical stability than equivalent SLN formulations, while resveratrol retained higher antioxidant activity when incorporated into NLCs than into SLNs in comparative dermal studies [203,204].

SLNs and NLCs enhance skin delivery mainly through occlusion, increased stratum corneum hydration, intimate contact with skin furrows, and possible follicular entry [201,205]. NLCs may achieve deeper cutaneous distribution than SLNs in selected models, with confocal microscopy showing greater localization in viable epidermis and, in some systems, dermal compartments [205]. Resveratrol-loaded NLCs showed approximately threefold higher dermal deposition than conventional carbopol gel, and NLCs enhanced skin retention of ferulic and vanillic acids compared with free actives [204,206].

The lipid matrix can be engineered for sustained, biphasic, or depot-like release, while surface modification with polyethylene glycol, hyaluronic acid, or lectins may increase residence time or direct particles toward selected keratinocyte populations [202]. Although SLNs and NLCs are often formulated from physiological or food-grade lipids and non-ionic surfactants, long-term stability should monitor lipid polymorphism, drug expulsion, particle size changes, and burst release [201,202]. Particles in the 100–300 nm range generally remain confined to the outer stratum corneum and follicular infundibulum in intact skin, whereas smaller particles or compromised barriers may increase translocation risk [202].

### 5.3. Polymeric Nanocarriers and Nanoemulsions

Polymeric nanocarriers include nanospheres, nanocapsules, and micelles prepared from synthetic biodegradable polymers such as poly (lactic-co-glycolic acid) (PLGA), polycaprolactone (PCL), and polylactic acid (PLA), or natural biopolymers such as chitosan [207]. PLGA is widely used because its lactide ratio determines hydrolysis rate and allows release to be tuned from days to months [208]. Chitosan provides a positive surface charge that promotes bioadhesion to negatively charged skin surfaces and may enhance permeation through electrostatic interactions with lipid head groups [209].

Polymeric encapsulation may improve the stability and controlled release of oxidation-prone or poorly soluble actives such as curcumin, retinoids, resveratrol, and EGCG, although this benefit remains formulation-specific [208,210,211]. In intact skin, particles of approximately 100–300 nm tend to accumulate through the transfollicular route, whereas barrier-impaired skin may permit greater deposition [207,212]. Confocal microscopy has shown limited PLGA nanoparticle accumulation in healthy skin but enhanced deposition in lesional atopic dermatitis skin, demonstrating that clinically impaired barrier states may alter nanocarrier distribution [212]. However, findings from inflammatory dermatoses should not be directly extrapolated to chronological aging or photoaging. Microneedle-assisted PLGA nanoparticle delivery can bypass the stratum corneum and distribute particles into epidermal and dermal microchannels [213].

Surface functionalization, including hyaluronic acid–CD44 or peptide–integrin targeting, is mechanistically plausible but remains largely translational rather than clinically validated for anti-aging outcomes [207,212,214]. Stimulus-responsive release from pH- or redox-sensitive polymers may provide additional temporal specificity in inflamed or UV-stressed skin, but most evidence remains preclinical or ex vivo [207,212,214]. Safety assessment should include polymer molecular weight, residual solvents, particle size, surface charge, penetration depth, and barrier status, because biomedical acceptance of polymers such as PLGA cannot be directly extrapolated to repeated cosmetic facial use [199,208,212].

Nanoemulsions are kinetically stable oil-in-water dispersions with droplet diameters generally between 10 and 200 nm, produced by high- or low-energy emulsification methods [215]. Their performance depends on oil phase selection, surfactant/cosurfactant composition, hydrophilic–lipophilic balance, aqueous phase properties, and residence time on the skin [215]. CoQ10 and resveratrol nanoemulsions have shown preserved antioxidant activity and improved stability compared with free formulations, while biosurfactant-stabilized bakuchiol nanoemulsions offer a synthetic surfactant-free strategy [216,217,218].

Nanoemulsions may enhance stratum corneum penetration by fluidizing intercellular lipid domains and improving partitioning of lipophilic actives into the skin [216,217]. Resveratrol-loaded nanoemulsion gels showed deeper deposition than conventional gels in ex vivo and instrumental studies, although these endpoints do not yet prove clinical anti-aging efficacy [217]. Co-delivery systems are feasible, as illustrated by paeonol/madecassoside nanoemulsions that improved barrier repair and anti-inflammatory endpoints in experimental UVB models [219]. Toxicological concerns include surfactant-mediated irritation, uncertain behavior in barrier-impaired skin, and regulatory documentation for droplets within the nanomaterial range [215,218].

### 5.4. Encapsulation of Probiotics, Postbiotics, and Microbiome-Active Compounds

Encapsulation is especially relevant for microbiome-directed formulations because live bacteria must remain viable and metabolically active despite exposure to heat, pH changes, osmotic stress, preservatives, oxygen, and UV light [220,221]. For topical live-biotic systems, encapsulation is therefore a key enabling technology, although most available data remain formulation-focused rather than clinically anti-aging oriented [220,221]. Postbiotics present fewer formulation constraints because they do not require viability maintenance, but bacterial lysates, SCFAs, exopolysaccharides, and bacteriocins still require protection against oxidation, temperature changes, UV light, and pH-mediated degradation [129,222].

Alginate-based systems are commonly used because calcium crosslinking forms a three-dimensional hydrogel network that protects entrapped bacteria from external stressors [220]. *Lactobacillus casei* encapsulated in alginate–tapioca flour microspheres and coated with selected biopolymers showed improved viability characteristics, although these findings support technological feasibility rather than clinical anti-aging efficacy [220].

Because intact bacteria are too large to cross the stratum corneum, topical probiotic effects are expected to occur through surface microbiome modulation, competitive exclusion, paracrine signaling, and interactions at follicular openings rather than dermal colonization. Alginate–prebiotic microspheres loaded with *L. casei* showed anti-pathogen activity and acceptable cytocompatibility in keratinocyte and fibroblast models [220,221]. For postbiotic butyrate and related small molecules, nanocarrier encapsulation is more compatible with delivery toward viable epidermal targets than live bacterial encapsulation [222].

Transfollicular delivery may be particularly relevant because the pilosebaceous unit represents an important niche for microbial modulation [220]. Hyaluronic acid-coated alginate-tapioca microspheres showed strong bioadhesion to skin-related cell surfaces, supporting the feasibility of targeted topical live-biotic carriers [220]. Co-encapsulation of microbial or postbiotic actives with prebiotic substrates reproduces the synbiotic principle at the formulation level, while stimuli-responsive postbiotic carriers remain promising but largely preclinical [223].

Safety concerns differ between probiotics and postbiotics. Live probiotic formulations require consideration of opportunistic infection risk, horizontal gene transfer, strain characterization, and unintended modification of the resident skin microbiome. These risks may be reduced by using well-characterized GRAS-status strains such as *L. plantarum*, *L. rhamnosus*, and *B. longum*. Postbiotics are generally easier to regulate as inanimate bacterial components, and topical formulations containing LactoSporin or *Bifidobacterium longum lysate* have been used in human studies without reported adverse effects [140,224]. Preservative compatibility remains a practical challenge for live biotic formulations and must be optimized on a product-specific basis [220].

### 5.5. Controlled and Stimuli-Responsive Release Systems

Most nanocarriers rely mainly on passive diffusion, whereas stimuli-responsive systems are designed to release cargo in response to local microenvironmental triggers such as pH, redox/ROS status, temperature, or enzyme activity [223,225]. These systems are conceptually attractive for anti-aging because photoaged skin is characterized by oxidative stress, inflammation, pH changes, enzymatic remodeling, and barrier disruption [223,225]. However, direct clinical evidence in cutaneous anti-aging remains sparse, and the field is still dominated by formulation, ex vivo, and preclinical studies [223,225].

pH-responsive systems use ionizable polymers, pH-labile bonds, or acid-responsive lipids to swell, dissolve, or disassemble under selected pH conditions [223,225]. Chitosan-based nanoparticles additionally exploit amine-group protonation, combining skin adhesion with controlled-release properties [223,225]. Thermoresponsive systems, including poly(N-isopropylacrylamide) (PNIPAM)-based platforms, undergo temperature-dependent structural transitions, but their non-biodegradability and limited dermatologic validation require caution [223,225]. ROS- and redox-responsive nanocarriers incorporate disulfide bonds, thioketal linkers, boronate esters, or selenium-containing groups to release cargo preferentially in oxidatively stressed environments [226]. Redox-responsive core-multishell nanocarriers have demonstrated trigger-dependent release and enhanced epidermal or dermal delivery compared with non-sensitive controls in skin models [226].

Enzyme-responsive systems are relevant because photoaged skin overexpresses MMPs, but MMP-cleavable nanocarriers remain largely extrapolated from smart drug-delivery research [223,225]. Multi-stimuli systems combining pH, ROS, temperature, or enzyme responsiveness may offer higher specificity, but they also increase synthesis, characterization, toxicology, and regulatory complexity [223,225]. Stability concerns include oxygen sensitivity for ROS-responsive systems, buffering requirements for pH-responsive systems, and temperature control for thermoresponsive platforms [223,225]. Overall, stimuli-responsive systems should be presented as future-facing precision tools rather than clinically validated anti-aging technologies [223,225,226].

### 5.6. The Epidermis–Dermis Penetration Gap and the Influence of Aging-Skin Phenotype

A central limitation across topical nanocarrier platforms is the epidermis–dermis penetration gap. Most rigid nanoparticles accumulate predominantly in the stratum corneum and follicular infundibulum of intact healthy skin, while only limited quantities reach the viable epidermis and dermis [213]. This is highly relevant to anti-aging because many clinically important targets, including fibroblast collagen production, MMP-driven ECM remodeling, and dermal vascularity, are located in the papillary and reticular dermis.

Confocal microscopy and quantitative studies show that even microneedle-assisted PLGA nanoparticle delivery deposits more particles in the epidermis than in the dermis [213]. An in silico model based on 103 published nanoparticle skin penetration studies confirmed that passive transcutaneous penetration of rigid nanoparticles larger than 10 nm is uncommon in intact skin, with follicular accumulation representing the dominant distribution pathway for many carrier types [227]. Conventional topical application, even with optimized nanocarriers, most reliably delivers actives to the stratum corneum and upper viable epidermis, whereas dermal access usually requires ultra-deformable vesicles, microneedles, sonophoresis, active penetration enhancement, or barrier compromise [192,212,213,227].

Chronologically aged and photoaged skin should not be considered interchangeable delivery environments. Chronologically aged, non-photoexposed skin is characterized by progressive changes in stratum corneum hydration, lipid organization, surface pH, sebum production, and barrier recovery capacity. However, these alterations do not necessarily imply uniformly increased baseline permeability or reliable passive access of nanoparticles to the viable epidermis or dermis. Permeability-related outcomes are influenced by anatomical site, sex, environmental conditions, formulation properties, and the specific method used to assess barrier function [228,229,230]. Therefore, in chronologically aged skin, topical nanocarrier strategies should primarily prioritize superficial deposition, barrier lipid restoration, hydration support, antioxidant protection, and microbiome-directed activity rather than unproven dermal delivery.

Photoaged skin presents a partially distinct scenario because chronic UV exposure may further impair stratum corneum cohesion, permeability barrier homeostasis, lipid organization, and inflammatory balance. Experimental studies suggest that UV -damaged skin may exhibit altered penetration of soft lipid and polymeric nanocarriers compared with non-irradiated skin [228,229,231]. Nevertheless, photoaging should not be interpreted as a condition in which dermal delivery occurs automatically. The available evidence supports altered or enhanced delivery under selected experimental conditions, but direct comparative studies in naturally photoaged human facial skin remain limited.

Accordingly, application scenarios should be differentiated. In chronologically aged skin, conventional liposomes, SLNs, NLCs, and nanoemulsions may be most appropriate for surface and stratum corneum-directed delivery of barrier lipids, antioxidants, and postbiotic or microbiome-supporting compounds. In photoaged skin, ultra-deformable vesicles and selected responsive systems may be considered for epidermal delivery of antioxidants, polyphenols, retinoid-like compounds, or peptides targeting oxidative stress, inflammation, and MMP-related pathways. In contrast, strategies intended to influence dermal fibroblasts or ECM remodeling should rely on validated assisted-delivery approaches, such as microneedles, rather than passive topical penetration alone [192,193,213,227].

Importantly, clinically barrier-impaired skin, including inflammatory dermatoses, should not be considered a surrogate for either chronological aging or photoaging. Although barrier disruption may increase nanocarrier deposition, it may also increase irritation, systemic exposure, and formulation-related safety concerns.

Therefore, studies of anti-aging nanocarriers should include validated imaging and quantification methods, such as confocal laser scanning microscopy, two-photon microscopy, optical coherence tomography, or chromatographic tissue analysis, to confirm actual delivery depth [207,213,227].

### 5.7. Layer-Specific Targeting and Carrier–Cargo Matching Framework

No single nanocarrier platform is universally superior; the optimal design should be selected according to the physicochemical properties of the active compound, the intended biological target, the required depth of delivery, and the safety profile of the recipient skin. A rational anti-aging formulation should therefore begin with the target skin compartment rather than with the carrier platform alone. The principal target compartments include the skin surface and stratum corneum, the follicular–pilosebaceous unit, the viable epidermis, and the superficial dermis.

Table 4 first compares the major delivery platforms according to cargo compatibility, expected target compartment, highest available evidence relevant to anti-aging application, and manufacturing scalability. Table 5 then complements this platform-based overview by summarizing the proposed penetration mechanisms and the experimental evidence supporting the expected deposition profile of each carrier system. Finally, Table 6 translates these platform and penetration data into a target-centric framework by matching skin compartments, aging-related objectives, bioactive cargo, delivery schemes, and principal limitations. Interpreted together, Table 4, Table 5 and Table 6 indicate that carrier selection should be driven by the intended biological target and the required skin compartment, rather than by particle size or technological novelty alone. Critically, these tables should not be interpreted as evidence that one carrier class is universally superior or that experimental deposition automatically translates into clinical anti-aging efficacy.

**Figure 2:**
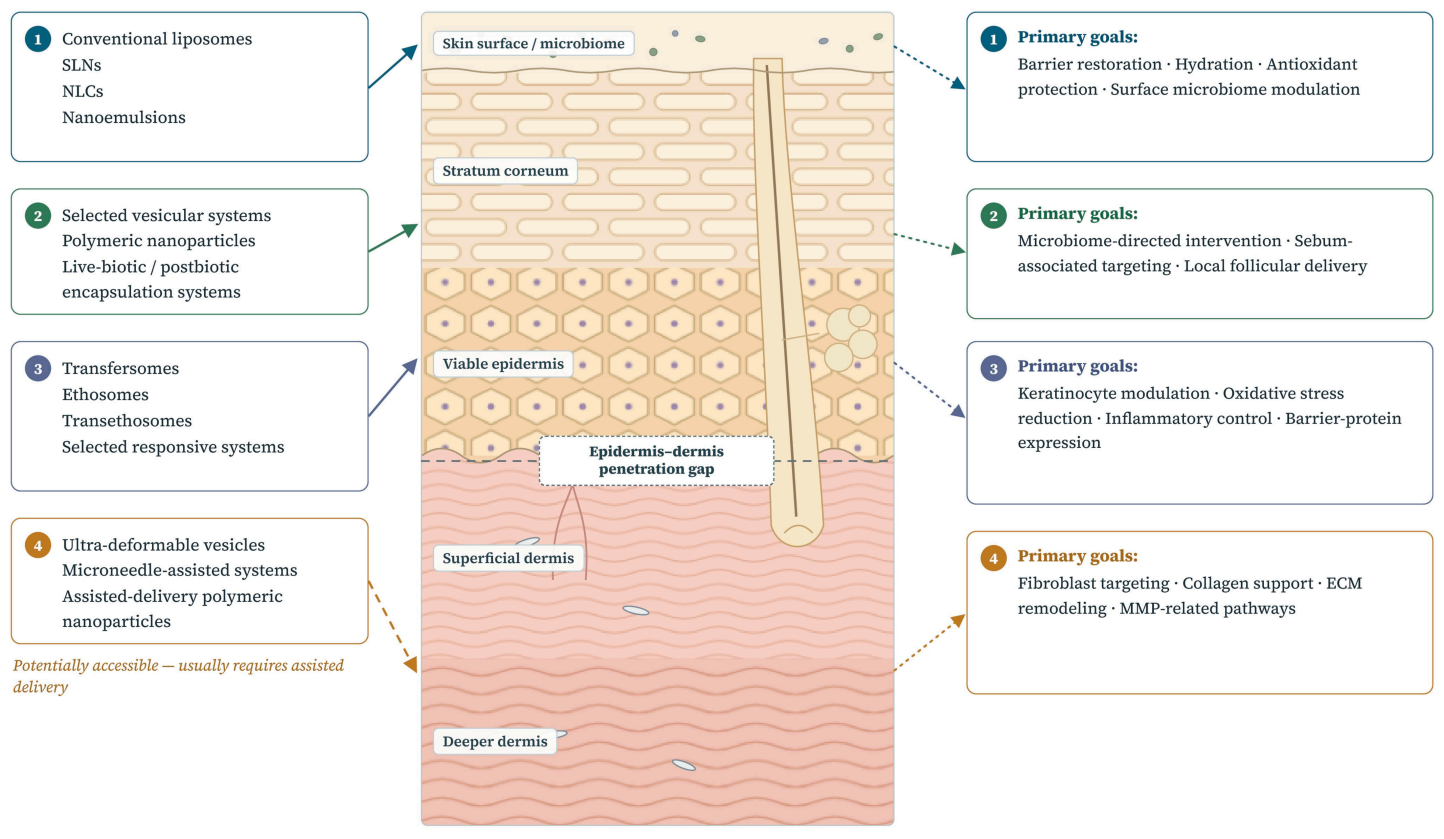
Layer-specific targeting and the epidermis–dermis penetration gap in anti-aging nanodelivery. A schematic representation of the principal cutaneous compartments relevant to anti-aging delivery: the skin surface and stratum corneum, follicular–pilosebaceous unit, viable epidermis, and superficial dermis. Conventional lipid-based systems are primarily suited for superficial deposition and barrier-directed delivery, whereas selected vesicular, polymeric, and responsive carriers may improve follicular or viable epidermal access. Reliable passive dermal delivery remains limited in intact skin and generally requires validated assisted-delivery approaches. Improved formulation performance or experimental deposition should not be interpreted as proof of clinically meaningful anti-aging efficacy. The figure is an original schematic prepared by the authors using vector-based HTML graphics and exported as a high-resolution image. It synthesizes the delivery mechanisms, target compartments, and penetration limitations discussed in Section 5.1, Section 5.2, Section 5.3, Section 5.4, Section 5.5, Section 5.6 and Section 5.7 and Table 4, Table 5 and Table 6 and was not adapted or reproduced from a previously published figure.

The anatomical basis for interpreting these penetration profiles and their layer-specific applications, including the epidermis–dermis penetration gap, is illustrated in Figure 2.

Figure 2 emphasizes that passive topical nanocarriers predominantly support superficial, follicular, or viable epidermal delivery, whereas reliable dermal targeting generally requires validated assisted-delivery approaches. This distinction is essential when interpreting nanocarrier performance in anti-aging applications, because improved formulation stability or experimental deposition does not necessarily imply clinically meaningful dermal efficacy.

At the skin surface and within the stratum corneum, the main therapeutic objectives are restoration of lipid organization, reduction in transepidermal water loss, antioxidant protection, and modulation of the surface microbial ecosystem. For these indications, excessive penetration is neither necessary nor desirable. Conventional liposomes, SLNs, NLCs, and nanoemulsions are particularly appropriate for improving the stability, retention, and local delivery of lipophilic antioxidants and barrier-supporting lipids within superficial skin compartments, although some NLC formulations may reach viable epidermal layers in selected models [187,192,201,202,203,204,205,206,215,216,217,218,219].

The follicular–pilosebaceous unit represents a distinct target for microbiome-directed and sebum-associated interventions. Follicular accumulation may be advantageous for carriers intended to act at local microbial niches or around the follicular infundibulum. Selected vesicular systems, polymeric nanoparticles, and alginate-based live biotic microspheres may be suitable for this purpose [195,207,220,221]. However, intact bacteria are expected to remain at the skin surface or follicular openings rather than penetrate into viable skin [220,221].

Delivery to the viable epidermis is more relevant for interventions targeting keratinocyte differentiation, oxidative stress, inflammatory signaling, barrier protein expression, and epidermal repair. Ultra-deformable vesicles, including transfersomes, ethosomes, and transethosomes, may improve access to viable epidermal compartments for selected polyphenols, antioxidants, peptides, and retinoid-like compounds [187,191,192,193,194,196,197]. Nevertheless, the extent of epidermal delivery remains formulation- and skin-condition-dependent, and ethanol-containing systems require particular caution in sensitive or barrier-impaired skin [192,193,200].

The superficial dermis is the most challenging target because fibroblasts, collagen synthesis, elastin organization, and matrix metalloproteinase activity are located beyond the principal barrier function of the epidermis. Passive topical delivery of rigid nanoparticles to the dermis is limited in intact skin [213,227]. Therefore, dermal targeting should be considered mainly for ultra-deformable vesicles, microneedle-assisted systems, or approaches used in barrier-impaired skin [192,193,212,213,227]. Such strategies require careful assessment of delivery depth, irritation potential, and possible systemic exposure.

Stimuli-responsive systems should be regarded as an additional release-control strategy rather than as an independent means of reaching a specific skin layer. Their value depends on combining a trigger-responsive material with a carrier capable of accessing the intended compartment. ROS-, pH-, temperature-, or enzyme-responsive systems may be relevant to cutaneous microenvironments characterized by oxidative stress, inflammation, altered pH, or matrix remodeling; however, direct clinical evidence in skin aging remains sparse [223,224,225,226]. The layer-specific matching of skin aging scenarios, therapeutic objectives, appropriate cargo, and delivery schemes is summarized in Table 6.

A key limitation across all platforms is the scarcity of head-to-head randomized controlled trials in photoaged human skin comparing different nanocarrier systems delivering the same active compound. Most available evidence is derived from ex vivo skin penetration studies, reconstructed or murine photoaging models, formulation studies, and small product-specific human trials using heterogeneous endpoints [140,198,224,227]. Improved encapsulation efficiency, skin retention, or experimental deposition should therefore not be equated with improvements in wrinkles, elasticity, pigmentation, barrier function, or patient-reported skin quality. Robust clinical claims should be reserved for systems supported by controlled human evidence, whereas most nanocarrier technologies should be considered promising translational platforms requiring independent replication [140,198,224].

Future studies should directly compare free and encapsulated cargo and report particle size, surface charge, carrier composition, loading efficiency, release profile, storage stability, and validated measures of deposition depth. Repeated-use safety, irritation potential, microbiome effects, and possible systemic exposure should also be assessed. Harmonized clinical outcomes, including three-dimensional wrinkle volumetry, high-frequency ultrasound, optical coherence tomography, barrier-related parameters, standardized photography, and patient-reported outcomes, are needed to enable meaningful inter-study comparisons. Manufacturing reproducibility, scalability, and variation in regulatory requirements across jurisdictions remain additional barriers to clinical translation.

An integrated comparison of the principal bioactive strategies and delivery approaches, including their mechanisms, evidence maturity, advantages, translational limitations, and current development status, is provided in Table 7.

Table 7 reinforces that the most appropriate delivery approach depends on the bioactive cargo, target compartment, level of supporting evidence, and translational limitation, rather than on carrier novelty alone.

Thus, delivery technology should be considered an enabling component of an anti-aging strategy rather than an independent determinant of efficacy. The clinical relevance of a carrier depends on the biological rationale of the cargo, the intended skin compartment, validated delivery depth, safety, and evidence of meaningful benefit in human studies. The following section addresses the translational and clinical implications of integrating these elements.

## 6. Integrated Strategies: Topical and Systemic Rejuvenation

Skin aging is a multidimensional process that cannot be adequately addressed by a single compound or delivery route acting in isolation. Integrated anti-aging strategies should therefore combine topical and systemic interventions when mechanistic complementarity is plausible, while recognizing that true clinical synergy remains incompletely demonstrated for many combinations [232]. This chapter synthesizes four strategic axes: nutricosmetic–cosmeceutical synergy, hormone–microbiome–skin modulation, combined ECM and antioxidant targeting, and age- or sex-specific personalization [232].

### 6.1. Rationale for Combined Topical–Systemic Strategies

The “inside–out + outside–in” rationale is based on spatial and pharmacokinetic complementarity. Oral interventions may influence systemic redox status, inflammatory tone, endocrine–metabolic signaling, and dermal fibroblast exposure, whereas topical actives can deliver higher local concentrations at the stratum corneum–epidermis interface [232]. However, robust head-to-head trials comparing oral-only, topical-only, and combined regimens remain limited; therefore, dual-route programs should be framed as mechanistically plausible rather than consistently superior [232].

Photoprotection is currently the most clinically developed model of topical–systemic integration. Combined oral plus topical standardized *Polypodium leucotomos* extract (PLE) was superior to topical PLE alone in patients with actinic keratoses, while systematic evidence supports oral PLE as an adjuvant photoprotective agent but emphasizes heterogeneity and the need for independent trials [171,233]. Astaxanthin has biologically plausible antioxidant and photoprotective activity, although much evidence remains mechanistic or derived from small clinical studies [168]. A topical combination of vitamin C, astaxanthin, and fermented turmeric improved fine lines, wrinkles, radiance, and hyperpigmentation in an open-label study, but the absence of placebo control limits causal interpretation [234]. A 2025 systematic review and meta-analysis of 40 randomized controlled trials found measurable benefits of dietary supplements on photoaging outcomes, supporting oral supplementation as an adjunctive approach rather than proving generalized oral–topical synergy [235].

### 6.2. Evidence-Supported Combination Models

Oral collagen peptides and topical signal peptides address collagen homeostasis through distinct but potentially convergent mechanisms. Oral collagen-derived peptides such as Pro-Hyp and Hyp-Gly can appear in the circulation after ingestion and may influence fibroblast activity and ECM remodeling, while preclinical studies support modulation of TGF-β/Smad signaling in photoaged skin models [81,236]. Topical signal peptides such as Pal-KTTKS and GHK/GHK-Cu have supportive but heterogeneous evidence and should not be presented as equivalent to retinoids in level of evidence [156,158]. A systematic review of randomized controlled trials reported improvements in hydration and elasticity with oral hydrolyzed collagen, but a more recent meta-analysis found that significant benefits were largely confined to industry-funded studies [161,237]. The randomized controlled trial by Reilly et al. supports a hydrolyzed collagen plus vitamin C supplement using clinical, biophysical, and imaging endpoints, but remains product-specific and requires independent replication [238].

Microbiome-directed combinations are consistent with the gut–skin axis model, although evidence remains stronger for barrier and inflammatory outcomes than for validated anti-aging endpoints. Oral synbiotics, such as fermented milk containing *Bifidobacterium breve* strain Yakult plus galacto-oligosaccharides, improved dry skin parameters and reduced gut-derived phenolic metabolites in healthy adult women [141,142]. These findings support the concept that microbial metabolites, immune modulation, and barrier-related pathways may influence cutaneous homeostasis [239]. Topical postbiotics act more directly at the skin surface, as Bifidobacterium longum lysate improved reactive skin parameters and 2% LactoSporin cream reduced visible wrinkle parameters in a small placebo-controlled study, although secondary biophysical outcomes were less consistent [140,224].

Nanocarriers may support integrated regimens by improving the stability, penetration, and compartment-specific delivery of labile antioxidants, retinoids, and peptides, but they should be considered pharmacotechnological enablers rather than clinically validated rejuvenation strategies. Nanoliposome co-delivery of carnosine, palmitoyl tripeptide-5, and acetyl hexapeptide-3 enhanced peptide delivery and anti-aging signals in experimental models, while hydroxypinacolone retinoate–carnosine nanoliposomes suggest improved epidermal or dermal delivery and multifunctional effects [240,241]. Reviews of smart nanocarriers support the conceptual value of pH-, thermo-, and ROS-responsive delivery, but emphasize the need for safety, stability, and long-term clinical validation [223].

### 6.3. Hormone–Microbiome–Skin Personalization

Hormonal decline, particularly postmenopausal estrogen deficiency, affects dermal thickness, collagen metabolism, vascularization, barrier lipid composition, and skin surface physiology. These endocrine changes may interact with age-related microbiome shifts, including altered microbial diversity, lipid substrate availability, pH, and beneficial microbial metabolites [239,242]. Dysbiosis may amplify inflammaging through innate immune activation and barrier disruption, but direct causal evidence linking menopausal microbiome changes to wrinkle progression remains limited [239,242].

Phytoestrogens, including genistein, daidzein, equol, and resveratrol, bind ERα and ERβ with variable affinity and have been associated with collagen stimulation, antioxidant activity, MMP modulation, and effects on melanogenesis in experimental models [243]. In postmenopausal women, a genistein-containing nutraceutical product combined with vitamin E, niacinamide, and ceramide improved hydration and barrier-related parameters compared with placebo, but clinical data remain formulation-specific and require caution in hormone-sensitive populations [151]. The equol phenotype illustrates microbiome-dependent nutraceutical response, as only approximately 30–50% of Western adults can convert daidzein into equol, a biologically active isoflavone metabolite with preferential ERβ-related activity [243]. This phenotype supports a precision-nutricosmetic hypothesis, but current evidence does not justify routine clinical prediction of skin aging response based on equol producer status alone [243].

Topical microbiome agents may complement systemic hormone-modulating strategies by targeting barrier lipids, sensory reactivity, and photo-inflammation. Topical *Streptococcus thermophilus* preparations increased stratum corneum ceramide levels, probably through bacterial sphingomyelinase activity [244,245]. *Bifidobacterium longum lysate* improved reactive skin parameters and may modulate sensory reactivity and barrier-related pathways [224]. Butyrate produced by commensal *Staphylococcus epidermidis* downregulated UVB-induced IL-6 through SCFA receptor signaling, supporting a mechanistic link between commensal metabolism and photo-inflammation [246]. These data support topical microbiome modulation, but do not yet prove synergy with systemic phytoestrogens.

Dietary microbiome modulation may also function as a systemic interface. Soy isoflavone-rich foods, galacto-oligosaccharides, inulin, and polyphenol-rich dietary patterns may support *Bifidobacterium*, *Lactobacillus*, and other metabolically favorable taxa, with downstream effects on SCFA production, gut barrier function, and systemic inflammatory tone [141,247]. Butyrate has plausible anti-inflammatory and barrier-supporting effects on keratinocytes, including histone deacetylase (HDAC)-related and lipid metabolism pathways [222]. Nevertheless, evidence that dietary microbiome modulation meaningfully improves estrogen-deficient wrinkling remains indirect, so this strategy should be presented as translational potential rather than established clinical efficacy [222].

### 6.4. ECM Stimulation, Antioxidant Protection, and Delivery Support

ECM degradation and oxidative stress form a self-reinforcing loop, as UV-induced ROS activate MAPK/AP-1 and NF-κB signaling, increase MMP-1, MMP-3, and MMP-9 expression, and impair TGF-β/Smad-driven procollagen synthesis [78,248]. Combined anabolic–antioxidant strategies are therefore mechanistically coherent, although clinical superiority over single-pathway regimens remains to be demonstrated in independent comparative trials [78,248].

Oral hydrolyzed collagen has meta-analytic support for hydration and elasticity, but interpretation is limited by industry funding and study heterogeneity [161,237]. Topical niacinamide has stronger dermatologic and cosmetic evidence for barrier improvement, pigmentation, sallowness, fine lines, and texture, while mechanistically supporting NAD+-dependent metabolism, ceramide synthesis, and anti-inflammatory effects [164,249]. Product-specific combinations, such as fermented fish collagen plus gamma-aminobutyric acid (GABA), are promising but should remain preliminary until independently replicated [250].

Antioxidant strategies should address multiple redox compartments without overstating evidence for complex “stacks.” Alpha-lipoic acid interacts with glutathione, vitamin C, and vitamin E redox networks [251]. Vitamin C is a hydrophilic antioxidant and essential collagen cofactor, but stability and formulation pH are critical [163]. Coenzyme Q10 supports mitochondrial redox biology, although human studies remain small [252,253]. Astaxanthin has membrane-related antioxidant and photoprotective plausibility, while ergothioneine is transported by organic cation transporter novel type 1 (OCTN1) and accumulates in mitochondria-rich tissues, although robust dermatologic randomized trial evidence remains limited [168,169].

The anti-catabolic component of this strategy involves suppression of UV-induced MMP signaling. Pal-KTTKS has split-face clinical evidence for wrinkle reduction, whereas GHK-Cu is supported mainly by mechanistic and cosmetic product research rather than large independent randomized controlled trials [156,158]. EGCG-containing transfersomes with hyaluronic acid suppressed UV-related oxidative and MMP pathways in experimental systems, but this should be interpreted as formulation and mechanistic evidence rather than proven clinical MMP inhibition [194]. Topical and oral PLE may reduce UV-induced oxidative and inflammatory signaling, but clinical anti-aging use should be framed mainly within photoprotection and actinic damage prevention [171,233].

Nanocarriers may enhance stability, skin retention, and local release in formulation, ex vivo, and preclinical studies; however, robust human evidence of anti-aging efficacy remains limited. Hydroxypinacolone retinoate (HPR)–carnosine nanoliposomes improved skin retention and multifunctional anti-aging readouts in experimental models [242]. Peptide co-delivery nanoliposomes have shown promising delivery and wrinkle-related signals, but the evidence remains formulation-driven and has not yet been independently reproduced in large dermatology randomized controlled trials [242]. Stimuli-responsive nanocarriers remain proof-of-concept systems for controlled delivery; direct clinical evidence in cutaneous aging is sparse, and long-term safety, standardization, and clinically meaningful endpoints remain important translational barriers [223].

### 6.5. Age- and Sex-Specific Strategy Design

Male skin is generally thicker, has higher collagen density, and produces more sebum than female skin, although these differences vary by anatomical site, age, and measurement method [254,255]. Female skin collagen loss accelerates after menopause, supporting the relevance of endocrine status for anti-aging strategy design [242]. These biological differences support a precision-medicine framework, but sex-specific recommendations should remain proportionate to the evidence and avoid deterministic assumptions [256].

In premenopausal women, cumulative photoexposure is usually more actionable than abrupt hormonal collagen loss; therefore, prevention should prioritize photoprotection, antioxidant defense, and barrier preservation. The Nambour randomized controlled trial demonstrated a 24% reduction in skin aging score with regular versus discretionary sunscreen use [257]. A 1-year prospective study in Brazilian women with phototypes II–VI found that twice-daily broad-spectrum sun protection factor (SPF) 60 sunscreen was associated with reduced progression of pigmentation and wrinkles [258]. Broad-spectrum sunscreen enriched with antioxidants may provide broader protection against UV-, visible-light-, pollution-, and infrared-A-related oxidative stress, although formulation-specific evidence remains important [259]. Topical bakuchiol 0.5% showed comparable improvement to retinol 0.5% for photoaging parameters with better tolerability in a small randomized controlled trial, and a systematic review supports its dermatologic potential while emphasizing limited trial numbers [260,261]. Oral PLE and astaxanthin may be considered adjunctive systemic photoprotection in selected contexts, while oral synbiotics remain promising but should not yet be considered core anti-aging standards [168,171,239].

## 7. Translational Challenges and Future Directions

Although bioactive compounds, microbiome-directed strategies, endocrine-modulating ingredients, and nanocarrier systems offer promising approaches for skin aging, their translation into clinically reliable interventions remains limited by several unresolved challenges. These include insufficient standardization of microbiome-based products, regulatory ambiguity between cosmetic and therapeutic claims, limited long-term safety data for endocrine-active compounds, incomplete understanding of nanocarrier toxicity, and the need for personalization according to hormonal, metabolic, and microbiome status.

### 7.1. Standardization of Microbiome Interventions

Microbiome-directed anti-aging strategies require greater standardization before they can be integrated into routine dermatologic or dermocosmetic practice [262]. For probiotics, clinical effects are strain-specific and cannot be extrapolated across species or genera; therefore, future studies should report strain identity, viability, dose, formulation matrix, storage conditions, and duration of use [262]. For postbiotics, standardization is equally important because bacterial lysates, metabolites, exopolysaccharides, and cell wall fragments may differ substantially in composition and biological activity [262]. Harmonized reporting of microbial composition, metabolite profiles, barrier-related outcomes, inflammatory markers, and clinical endpoints will be essential to distinguish true microbiome modulation from nonspecific cosmetic benefit [262].

### 7.2. Regulatory Complexity: Cosmetic Versus Therapeutic Claims

A major translational barrier is the distinction between cosmetic and therapeutic claims. Products intended to improve hydration, radiance, texture, or the appearance of wrinkles are generally positioned as cosmetics, whereas claims involving inflammation, wound healing, immune modulation, hormone regulation, or treatment of barrier dysfunction may approach therapeutic territory [263]. This distinction is particularly relevant for bioactive compounds with endocrine, immunologic, or microbiome-modulating activity. Future product development should therefore align mechanistic claims with regulatory category, level of evidence, and safety documentation, while avoiding overstated claims based mainly on in vitro, ex vivo, animal, or short-term product-specific studies [263].

### 7.3. Safety of Long-Term Endocrine-Modulating Ingredients

Endocrine-modulating ingredients in dermatologic and dermocosmetic products should not be regarded as purely local interventions. The skin is a hormonally responsive organ connected to systemic estrogenic, androgenic, glucocorticoid, and metabolic signaling pathways, and long-term use raises questions regarding cumulative biological exposure, particularly in hormonally vulnerable patients [21,95].

Caution is especially warranted in hormone-sensitive populations, including women with a history of estrogen-dependent breast or endometrial cancer, patients with clinically significant hyperandrogenic disorders, and individuals already receiving systemic menopausal hormone therapy. Although hormone-based strategies may improve skin or mucosal symptoms, systemic hormone therapy should not be prescribed solely for cutaneous anti-aging benefit; skin improvement should instead be considered a possible secondary benefit of appropriately indicated menopausal care [21,264].

Cumulative exposure also remains relevant. Repeated application over large body surface areas, prolonged treatment duration, lipophilic formulations, occlusion, and impaired barrier function may increase systemic absorption beyond what is expected for a local product [21,95]. This is particularly important for estrogenic approaches, because restoring estrogen signaling in aged skin is biologically attractive, but safe long-term cutaneous estrogen targeting remains incompletely resolved [95]. Therefore, endocrine-modulating ingredients should be presented as biologically plausible and potentially useful, but not definitively risk-free [95,264].

### 7.4. Nanocarrier Safety, Manufacturing, Regulatory Requirements, and Clinical Implementation

Nanocarrier-based anti-aging strategies require careful toxicological assessment because improved delivery may also increase exposure of viable epidermal or dermal compartments to active compounds and excipients. Safety evaluation should consider particle size, surface charge, lipid or polymer composition, surfactant concentration, degradation products, residual solvents, penetration depth, and behavior in barrier-impaired skin [265]. This is particularly important in aged, photoaged, inflamed, or dermatitic skin, where barrier disruption may enhance penetration and increase irritation or systemic exposure.

Long-term safety data remain limited for many cosmetic nanocarriers, especially stimuli-responsive systems, polymeric nanoparticles, and complex multi-active formulations [266]. Although liposomes, solid lipid nanoparticles, nanostructured lipid carriers, nanoemulsions, and polymeric systems may improve stability and cutaneous retention, clinical anti-aging claims should distinguish between enhanced formulation performance and proven biological rejuvenation. Future studies should include standardized physicochemical characterization, validated penetration imaging, repeated-use irritation testing, and long-term safety monitoring.

Beyond toxicology, clinical translation requires reproducible scale-up, preservation of critical carrier characteristics—including particle size, surface charge, encapsulation efficiency, release profile, and shelf life—and regulatory alignment with the intended product claim. Improved skin retention or experimental penetration should not be considered sufficient for clinical implementation without controlled human trials demonstrating clinically meaningful benefit, repeated-use safety, and, where relevant, local or systemic exposure [140,198,224,238].

### 7.5. Personalization Based on Hormonal, Metabolic, and Microbiome Status

A dermato-endocrinologic approach to skin aging should distinguish between premenopause, perimenopause, and postmenopause, because the endocrine drivers of cutaneous aging differ across these stages. In premenopausal women, ovarian estrogen production is usually preserved, and skin aging is more often driven by photoexposure, oxidative stress, lifestyle factors, and metabolic inflammation than by hormone deficiency [21]. Prevention should therefore prioritize photoprotection, antioxidant support, barrier maintenance, and correction of insulin-resistant or inflammatory phenotypes rather than hormone-directed therapy [21].

Perimenopause is a dynamic and clinically underrecognized stage, characterized by fluctuating estradiol levels, progressive ovulatory dysfunction, and declining progesterone production before sustained hypoestrogenism is established [21,267]. This is often the period when patients first report dryness, reduced elasticity, barrier discomfort, altered sebaceous activity, or early hair changes [21,267]. When systemic hormone therapy is indicated for standard menopausal reasons, stabilization of the endocrine milieu may indirectly improve skin quality, but cutaneous benefit should remain a secondary consideration [21,267].

Postmenopause is characterized by ovarian estrogen deprivation, a shift toward weaker peripheral estrogen production, and a decline in androgenic precursors. This hormonal environment is associated with dermal thinning, reduced vascularity, impaired wound healing, dryness, and progressive loss of structural support [21,95,264]. Meta-analytic data suggest that menopausal hormone therapy may improve skin elasticity, thickness, and collagen content, but estrogen-centered strategies should be considered only in appropriately selected patients and within the broader framework of menopausal care [21,95,264].

Androgen shifts also deserve attention, particularly during peri- and postmenopause, when declining estrogen may unmask relative androgenic effects even in the absence of biochemical hyperandrogenism [21,267,268,269]. This may help explain adult acne, seborrhea, facial hair changes, or patterned scalp hair thinning. Testosterone should not be presented as a standard anti-aging skin therapy, because its best supported postmenopausal indication relates to sexual function, while acne and hirsutism remain clinically relevant adverse effects [21,267,268,269].

In practical terms, personalization should be stage-specific and phenotype-driven. Premenopause calls for exposome- and metabolism-oriented prevention; perimenopause requires recognition of endocrine instability and symptom clustering; and postmenopause may justify carefully selected estrogen-centered strategies, always balanced against safety, metabolic profile, and hormone sensitivity [21,95,264]. Precision dermatology in this context does not mean prescribing more hormones, but applying the correct hormonal, metabolic, and microbiome framework to the correct biological stage.

Practical stratification may combine endocrine context, including age, sex, menopausal stage, and hormone-related symptoms; metabolic markers, such as adiposity, glycemic control, insulin resistance, lipid status, and inflammatory burden; and standardized microbiome and barrier measures. These variables should be interpreted alongside the clinical skin aging phenotype, including photoexposure, xerosis, sebaceous activity, transepidermal water loss, pigmentation, and wrinkle pattern. At present, they should be considered candidate stratification tools rather than validated predictors of response to anti-aging interventions [21,34,35,36,37,95,228,257].

### 7.6. Future Directions: From Mechanistic Promise to Clinical Implementation

Future research should move from broad anti-aging claims toward predefined, testable implementation pathways. First, microbiome-directed interventions require strain- and product-level selection rather than genus- or species-level generalization. For probiotics, future studies should report full strain identification, viability at the end of shelf life, dose, administration route, formulation matrix, storage conditions, and duration of exposure [128]. For postbiotics, the producing strain, manufacturing process, compositional fingerprint, active metabolite or cellular fraction, dose, and batch-to-batch reproducibility should be clearly reported [129,131]. Candidate strains or postbiotic preparations should be selected according to a predefined biological rationale, such as barrier support, the suppression of inflammatory signaling, the modulation of oxidative stress, enhancement in antimicrobial defense, or the production of relevant microbial metabolites [132,133,134].

Second, carrier optimization should be guided by the intended cutaneous compartment and cargo properties rather than by novelty alone. Surface- and stratum corneum-directed interventions should prioritize local retention, barrier compatibility, and controlled superficial release, whereas epidermal or dermal targets require validated evidence of delivery depth. Formulation development should systematically compare free and encapsulated cargo, carrier size, surface charge, lipid or polymer composition, loading efficiency, release kinetics, storage stability, and performance in intact and barrier-impaired skin [186,187,199,200,201,202,207,261]. For dermal fibroblast- or ECM-directed strategies, claims of dermal delivery should be supported by validated penetration studies and, where appropriate, assisted-delivery approaches rather than inferred from formulation characteristics alone [192,193,213,227].

Third, preclinical evaluation should be standardized before progression to human studies. Relevant models should include reconstructed human epidermis, ex vivo human skin, and, when appropriate, photoaging models that distinguish superficial deposition from viable epidermal or dermal delivery. Studies should report validated imaging or quantitative distribution methods, including confocal laser scanning microscopy, optical coherence tomography, tape stripping, or tissue drug quantification [198,213,227,261]. Repeated-use irritation testing, sensitization assessment, microbiome-perturbation analysis, and long-term safety monitoring are particularly important for live-biotic formulations, endocrine-active compounds, polymeric nanoparticles, and stimuli-responsive systems [199,200,223,261].

Finally, clinical studies should adopt harmonized designs and endpoints. Randomized controlled trials should clearly define the aging phenotype under study, including chronological aging, photoaging, menopausal skin change, xerosis, or microbiome-associated barrier impairment. Essential reporting variables should include age, sex, Fitzpatrick phototype, anatomical site, cumulative photoexposure, hormonal status, metabolic profile, baseline skin barrier status, microbiome profile where relevant, intervention composition, dose, and delivery platform. Core clinical outcomes should combine validated objective measures—such as three-dimensional wrinkle analysis, corneometry, transepidermal water loss, cutometry, high-frequency ultrasound, pigmentation assessment, and standardized photography—with patient-reported outcomes and adverse-event reporting. Studies of microbiome-directed products should additionally include transparent sequencing, bioinformatics, and metadata reporting, ideally aligned with STORMS guidance [228,257].

This framework would allow future studies to distinguish formulation performance from clinically meaningful anti-aging benefit, identify which biological subgroups are most likely to respond, and support a more credible transition from experimental platforms to evidence-based precision dermatology.

## 8. Conclusions

Skin aging is not driven by a single molecular defect, but by the progressive interaction of oxidative stress, mitochondrial dysfunction, cellular senescence, ECM degradation, inflammaging, barrier impairment, endocrine–metabolic dysregulation, and microbiome imbalance. This review highlights the endocrine–microbiome–skin axis as a useful integrative framework: hormonal changes influence sebaceous activity, lipid composition, hydration, barrier recovery, vascularization, and immune tone, thereby reshaping the cutaneous microbial niche. Conversely, microbial metabolites and microbial community structure may influence epithelial differentiation, inflammatory signaling, oxidative balance, barrier function, and systemic endocrine–metabolic pathways.

From a therapeutic perspective, the principal intervention targets are interconnected rather than independent. The preservation of collagen and elastin homeostasis, reduction in reactive oxygen species and mitochondrial dysfunction, restoration of epidermal lipids and barrier integrity, modulation of microbiome-related inflammation, and correction of endocrine–metabolic contributors may collectively improve skin resilience more effectively than single-pathway approaches. This supports a multidimensional strategy in which the biological mechanism, skin aging phenotype, and intended target compartment are considered together.

Microbiome-directed bioactive compounds—including probiotics, prebiotics, postbiotics, synbiotics, phytoestrogens, polyphenols, peptides, antioxidants, mitochondrial protectors, adaptogens, and metabolic modulators—show complementary mechanisms relevant to skin aging. However, their translational maturity is heterogeneous. Selected strain-specific probiotic, postbiotic, peptide, collagen, antioxidant, and phytoestrogen interventions have shown encouraging human results, whereas many proposed effects remain supported mainly by in vitro, ex vivo, animal, or product-specific studies. Therefore, biological activity should not be generalized across strains, doses, formulations, delivery routes, or compound classes.

Advanced delivery systems may improve the stability, retention, controlled release, and local availability of bioactive compounds, but carrier selection should be guided by the intended skin compartment. Surface- and stratum corneum-directed systems are most relevant for barrier restoration, hydration, antioxidant protection, and microbiome modulation; viable epidermal delivery may be appropriate for oxidative, inflammatory, and keratinocyte-related targets. In contrast, dermal fibroblast and ECM targets remain difficult to reach through passive topical delivery because of the epidermis–dermis penetration gap. Improved formulation performance or deeper experimental deposition should therefore not be interpreted as evidence of clinical rejuvenation without validated penetration studies and controlled human trials.

A substantial translational gap remains between laboratory promise and clinical implementation. Improved antioxidant activity, encapsulation efficiency, skin retention, or ex vivo penetration does not necessarily predict clinically meaningful improvement in wrinkles, elasticity, pigmentation, barrier function, or patient-reported skin quality. Many available studies are limited by simplified cell models, short-term animal experiments, non-standardized formulations, or heterogeneous product-specific human studies. In addition, biologically active interventions may be strain-, metabolite-, dose-, vehicle-, and batch-dependent, while nanocarrier performance may vary according to particle size, surface charge, composition, release profile, storage conditions, anatomical site, and the integrity of the recipient skin barrier. Repeated-use safety, microbiome perturbation, irritation potential, systemic exposure, manufacturing reproducibility, shelf life, regulatory classification, and cost-effective scale-up must also be addressed before laboratory findings can be translated into reliable clinical interventions.

Future progress will depend on implementation-oriented research. Priorities include strain- and postbiotic-level characterization; carrier–cargo optimization according to the target skin compartment; standardized assessment of penetration depth, repeated-use safety, and microbiome effects; and independently conducted clinical trials with harmonized biophysical, imaging, microbiome, and patient-reported outcomes. Stratification by chronological versus photoaged skin, age, sex, phototype, hormonal status, metabolic phenotype, barrier characteristics, and baseline microbiome profile will be important for identifying responders. Overall, microbiome-directed bioactive strategies combined with precision delivery technologies represent a promising but still evolving route toward evidence-based, personalized preservation of cutaneous homeostasis during aging.

## Figures and Tables

**Table 1:** Representative microbiome-targeted dermocosmetic and nutricosmetic platforms: terminology, evidence relevant to skin aging, and key limitations.

Intervention Category	Representative Ingredient or Platform	Route	Evidence Relevant to Skin Aging	Main Limitation
Strain-specific probiotics	*Lactobacillus plantarum* HY7714; *Bifidobacterium breve* M-16V	Oral	Controlled human studies reported improvements in selected wrinkle, elasticity, transepidermal water loss, VISIA, pigmentation, or pore-related outcomes [135,136]	Effects are strain-, dose-, population-, and formulation-specific
Heat-treated microbial preparation	Heat-treated *Latilactobacillus sakei* KABP-065	Oral	Controlled human study reported improved cheek elasticity in a subgroup of middle-aged women [137]	Requires clear compositional characterization and replication
Synbiotic formulation	*B. breve* Yakult plus galacto-oligosaccharides	Oral	Human studies reported improved skin-moisture parameters and changes in gut-derived phenolic metabolites [138,139]	Evidence is limited and not sufficient to establish generalized anti-aging efficacy
Microbial-derived topical ingredient	LactoSporin^®^ cream containing extracellular metabolite from *Bacillus coagulans*	Topical	One randomized controlled trial reported reductions in visible wrinkles and fine lines [140]	Product-specific evidence; not interchangeable with live probiotics
Bacterial lysate or ferment-derived ingredient	*Bifida ferment* lysate; *Lactobacillus* ferment-derived ingredients	Topical	In vitro barrier-supporting and immunomodulatory evidence [132]	Human anti-aging evidence remains insufficient or product-specific
Putative prebiotic ingredients	Inulin, fructo-oligosaccharides, galacto-oligosaccharides, alpha-glucan oligosaccharides	Topical or oral	Biological rationale for selective microbial support; limited direct anti-aging evidence	Must demonstrate selective utilization in the intended microbiome and a measurable host benefit

**Table 2:** Antioxidant and mitochondrial bioactive compounds for skin aging: mechanisms, delivery considerations, evidence hierarchy, and translational limitations. Evidence descriptions refer to the highest available study modality. Improved formulation performance or skin penetration should not be interpreted as proof of clinical anti-aging efficacy.

Compound	Main Mechanism Relevant to Skin Aging	Delivery Route/Formulation Relevance	Highest Level of Evidence and Study Modality	Main Translational Limitation	Key Supporting References
Vitamin C/L-ascorbic acid	Cofactor for prolyl- and lysyl-hydroxylases required for collagen post-translational modification and triple-helix stabilization; scavenges ROS and regenerates vitamin E.	Mainly topical; efficacy depends on acidic pH, adequate concentration, formulation stability, and use of stable derivatives such as magnesium ascorbyl phosphate, sodium ascorbyl phosphate, tetrahexyldecyl ascorbate, or ascorbyl glucoside.	Limited controlled human evidence; supported by mechanistic data.	Intrinsic instability, oxidation, pH sensitivity, and formulation-dependent penetration.	[163]
Niacinamide/nicotinamide	Precursor of NAD+ and Nicotinamide adenine dinucleotide phosphate (NADP+); supports mitochondrial bioenergetics, barrier function, cellular repair, ECM stimulation, anti-glycation activity, and pigmentation modulation.	Mainly topical; 5% niacinamide has been evaluated in clinical studies.	Controlled human clinical evidence.	Benefits are formulation- and concentration-dependent; effects are generally gradual and require sustained use.	[164]
Coenzyme Q10/ubiquinone	Mitochondrial electron carrier and lipophilic antioxidant; supports mitochondrial redox biology and may reduce ultraviolet A (UVA)-induced oxidative damage.	Topical and oral; topical formulations may improve epidermal energy metabolism and wrinkle depth, while oral supplementation may support systemic redox status.	Limited controlled human evidence; supported by mechanistic data.	Human anti-aging studies remain relatively limited and should be interpreted cautiously.	[165]
Tocotrienols and tocopherols/vitamin E family	Protect lipid-rich cutaneous compartments from peroxidation and may synergize with vitamin C in the cutaneous redox network.	Topical and oral potential; tocotrienols are of interest because of high membrane mobility and antioxidant activity in the stratum corneum.	Predominantly ex vivo/in vitro evidence.	Current tocotrienol data should not yet be interpreted as established anti-photoaging efficacy.	[17]
Astaxanthin	Marine xanthophyll carotenoid with high singlet-oxygen quenching capacity and photoprotective plausibility.	Oral and/or topical use has been investigated.	Limited controlled human evidence; supported by mechanistic data and repair.	Heterogeneity and variable study quality limit inference; larger independent randomized controlled trials are needed.	[167,168]
Ergothioneine	Sulfur-containing amino acid transported by organic cation transporter novel type 1/solute carrier family 22 member 4 (OCTN1/SLC22A4); scavenges hydroxyl radicals and singlet oxygen, chelates redox-active metals, and protects mitochondrial DNA from oxidative damage.	Mainly systemic/nutritional and potential topical antioxidant use.	Predominantly ex vivo/in vitro evidence.	Direct clinical evidence for cutaneous anti-aging endpoints remains limited.	[169]
α-Lipoic acid/thioctic acid	Amphipathic antioxidant active in both lipid and aqueous compartments; regenerates vitamins C and E and intracellular glutathione.	Topical; 5% α-lipoic acid has been evaluated clinically.	Limited controlled human evidence; supported by mechanistic data.	Evidence is based on limited clinical data; irritation and formulation tolerability should be considered.	[170]
Polypodium leucotomos extract	Oral hydrophilic fern extract with antioxidant and immunomodulatory activity; relevant to photoprotection and actinic-damage prevention.	Primarily oral systemic photoprotective adjuvant, often combined with topical sunscreen.	Controlled human clinical evidence for photoprotection; not established for generalized rejuvenation.	Evidence is strongest for photoprotection and actinic damage, not generalized rejuvenation.	[171,172]

**Table 3:** The major classes of microbiome-directed and multifunctional bioactive compounds for skin aging: mechanisms, targets, and evidence maturity. An overview of the principal bioactive classes discussed in Section 4, including probiotics, postbiotics, prebiotics/synbiotics, phytoestrogens, polyphenols, bioactive peptides, collagen-derived compounds, antioxidants/mitochondrial protectors, and adaptogens/metabolic modulators. The biological targets and evidence categories were synthesized by the authors from the studies and reviews cited in the corresponding subsections of the manuscript. The evidence categories reflect the highest available study modality and should not be interpreted as evidence of equivalent clinical efficacy across compounds, strains, formulations, doses, or delivery routes.

Bioactive Class	Main Biological Targets/Mechanisms	Evidence Maturity
Probiotics	Barrier support; microbiome modulation; anti-inflammatory signaling	Controlled human clinical evidence for selected strain-specific interventions
Postbiotics	Barrier support; antimicrobial defense; oxidative stress modulation	Limited controlled human evidence; supported by mechanistic data
Prebiotics/Synbiotics	Microbiome support; commensal balance; barrier homeostasis	Predominantly preclinical/mechanistic evidence
Phytoestrogens	Endocrine-responsive pathways; antioxidant effects; collagen support	Limited controlled human evidence
Polyphenols	Antioxidant activity; anti-inflammatory effects; MMP inhibition/ECM preservation	Limited controlled human evidence; strong mechanistic rationale
Bioactive peptides	Fibroblast stimulation; ECM synthesis; MMP modulation	Mixed evidence: controlled human and product-specific studies
Collagen-derived compounds	Hydration; elasticity; dermal matrix support	Controlled human clinical evidence
Antioxidants/mitochondrial protectors	ROS reduction; mitochondrial support; anti-photoaging	Heterogeneous evidence: some controlled human studies, substantial mechanistic support
Adaptogens/metabolic modulators	Stress response modulation; metabolic balance; anti-inflammatory support	Predominantly preclinical/exploratory evidence

**Table 4:** Comparative features, primary target compartments, and translational evidence of advanced anti-aging delivery platforms. Evidence descriptions refer to the highest available study modality. Improved formulation performance or skin penetration should not be interpreted as proof of clinical anti-aging efficacy.

Platform	Particle/Droplet Size	Ideal Active Class	Primary Target Compartment/Expected Penetration	Highest Available Evidence	Industrial Scalability
Liposomes/ultra-deformable vesicles	50–200 nm; conventional liposomes 20–150 nm; variable transfersomes	Peptides, polyphenols, retinoids, hydrophilic and amphiphilic actives	Viable epidermis; limited dermal delivery with ultra-deformable vesicles	Limited human/product studies; few robust head-to-head RCTs	High; extrusion, high-pressure homogenization, microfluidics
Solid lipid nanoparticles	50–300 nm	Lipophilic antioxidants, including CoQ10 and vitamin E; occlusion-dependent actives	Stratum corneum and upper epidermis	Limited; mostly in vitro and ex vivo evidence	Moderate; scalable by high-pressure homogenization and microemulsion methods
Nanostructured lipid carriers	100–300 nm	Lipophilic, oxidation-prone actives, including CoQ10, retinoids, and resveratrol	Viable epidermis; dermal delivery is model-dependent	Mostly in vitro, ex vivo, and preclinical evidence; limited human topical data	High; high-pressure homogenization and scalable lipid-processing methods
PLGA/polymeric nanoparticles	50–500 nm; polymeric micelles 20–80 nm	Retinoids, curcumin, polyphenols, and surface-targetable actives	Stratum corneum and follicular infundibulum with passive delivery; epidermis and dermis with microneedle assistance	Formulation and ex vivo evidence; dermal targeting requires barrier disruption or assisted delivery	Moderate to high; nanoprecipitation, emulsion, and solvent evaporation methods
Nanoemulsions	10–200 nm	Co-delivery of incompatible hydrophilic and lipophilic actives; bakuchiol, CoQ10, resveratrol	Stratum corneum and upper epidermis; clinical dermal delivery remains uncertain	In vitro and ex vivo evidence; clinical anti-aging RCTs remain limited	Very high; high-pressure homogenization, ultrasonication, and spontaneous emulsification
Encapsulated probiotics/postbiotics	Microspheres 50–500 μm; postbiotic nanocarriers 50–200 nm	Live bacteria, bacterial lysates, SCFAs, and exopolysaccharides	Stratum corneum and follicular orifice for live bacteria; viable epidermis possible for postbiotic nanocarriers	Selected postbiotic/lysate human data; live encapsulation mainly formulation-level evidence	Moderate; spray drying, extrusion, and alginate gelation

**Table 5:** Penetration mechanisms and layer-specific suitability of advanced delivery systems used for precision anti-aging targeting.

Carrier System	Primary Penetration Mechanism	Secondary Mechanism	Most Suitable Target Skin Compartment	Key Structural Feature	Evidence Modality
Conventional liposomes	Lipid bilayer fusion with stratum corneum intercellular lipids	Transfollicular accumulation	Stratum corneum, upper viable epidermis, and follicular openings	Phospholipid bilayer with aqueous core; usually 50–200 nm	Ex vivo and CLSM-based evidence
Transfersomes	Osmotically driven intact vesicle deformation through stratum corneum channels	Lipid bilayer fusion	Viable epidermis; limited superficial dermal delivery in selected formulations	Edge activator, such as sodium deoxycholate, conferring extreme elasticity	Ex vivo, in vitro, and formulation studies
Ethosomes	Ethanol-mediated stratum corneum lipid fluidization and vesicle deformation	Transient barrier disruption	Stratum corneum and viable epidermis	20–45% ethanol within phospholipid vesicles	Ex vivo studies and limited clinical translation
Transethosomes	Combined ethanol-mediated fluidization and edge activator-driven deformability	Osmotic gradient-driven penetration	Viable epidermis; limited superficial dermal delivery in selected models	Phospholipid vesicle containing both ethanol and edge activator	Ex vivo and formulation-level evidence
SLN/NLC	Occlusion-mediated reduction in TEWL and increased stratum corneum hydration gradient	Nanosize intimate contact with stratum corneum furrows and follicles	Stratum corneum, upper epidermis, and follicular infundibulum	Solid or semi-crystalline lipid matrix; NLCs contain a partially amorphous lipid phase	Ex vivo, preclinical, and limited in vivo evidence
PLGA/polymeric nanoparticles	Transfollicular accumulation, usually within the 100–300 nm range	Barrier-selective enhanced penetration in photoaged or damaged skin	Follicular infundibulum; viable epidermis and superficial dermis mainly with microneedle-assisted delivery	Biodegradable polymer matrix; surface ligands such as HA or RGD may be used for active targeting	CLSM, human skin models, and microneedle-assisted models
Nanoemulsions	Stratum corneum lipid fluidization through surfactant partitioning into intercellular domains	Maximized interfacial area and active concentration gradient at the stratum corneum	Stratum corneum and upper viable epidermis	Oil-in-water droplets, usually 10–200 nm, stabilized by surfactant/cosurfactant systems or secondary gel matrix	DSC, FTIR, CLSM, Franz diffusion, and ex vivo studies
Stimuli-responsive systems	Passive penetration according to the base carrier type	Triggered release in response to pH, ROS/redox status, MMPs, or temperature	Depends on the underlying carrier; release may be triggered within the epidermal or superficial dermal microenvironment	Labile bonds, LCST polymers, redox-sensitive linkers, or enzyme-cleavable structures	Ex vivo and preclinical evidence

**Table 6:** Layer-specific matching of skin targets, bioactive cargo, and nanodelivery schemes for anti-aging interventions.

Expected Primary Deposition Compartment	Relevant Aging-Skin Application Scenario	Main Aging-Related Objective	Appropriate Bioactive Cargo	Preferred Delivery Scheme	Main Limitation	Key Supporting References
Skin surface and stratum corneum	Chronologically aged, xerotic, low-sebum, or menopausal skin; prioritize barrier support and superficial microbiome modulation.	Barrier restoration, hydration, lipid replenishment, local antioxidant protection, surface microbiome support	CoQ10, vitamin E, lipophilic antioxidants, barrier lipids, postbiotic lysates, selected prebiotics	SLNs, NLCs, conventional liposomes, nanoemulsions	Deeper dermal delivery is not expected and is generally unnecessary	[187,201,202,203,204,205,206,215,216,217,218,219]
Follicular–pilosebaceous unit	Site-specific sebaceous or microbiome imbalance; relevant mainly for follicular and surface-directed interventions.	Local microbiome modulation, sebaceous balance, antioxidant protection, follicular inflammatory control	Live probiotics, postbiotics, prebiotic substrates, antimicrobials, sebum-modulating compounds	Polymeric nanoparticles, follicle-targeted vesicles, alginate microspheres, encapsulated microbiome-active systems	Live bacteria remain confined mainly to the surface and follicular opening	[195,207,220,221,227]
Viable epidermis	Photoaged or oxidatively stressed skin; antioxidant, anti-inflammatory, barrier protein, and epidermal repair targets.	Keratinocyte differentiation, barrier protein expression, reduction in oxidative stress and inflammaging	Peptides, polyphenols, retinoid-like compounds, antioxidants, small postbiotic metabolites	Transfersomes, ethosomes, transethosomes, selected NLCs, postbiotic nanocarriers	Penetration is formulation- and barrier status-dependent; ethanol-containing systems may irritate sensitive skin	[187,191,192,193,194,196,197]
Superficial dermis—potentially accessible with assisted delivery	Advanced photoaging or matrix-remodeling targets; potentially accessible only with assisted or validated enhanced-delivery approaches.	Fibroblast stimulation, collagen and elastin homeostasis, MMP modulation, reduction in oxidative matrix damage	Signal peptides, retinoid-like compounds, antioxidants, anti-glycation agents	Ultra-deformable vesicles, microneedle-assisted polymeric nanoparticles, assisted-delivery systems	Passive dermal delivery is limited in intact skin; safety and systemic exposure require assessment	[192,193,212,213,227]
Oxidative/inflammatory microenvironment within epidermis or superficial dermis	Photoaged or UV-stressed skin; predominantly preclinical application for triggered-release systems.	Triggered release in areas with elevated ROS, altered pH, or enzymatic remodeling	Antioxidants, anti-inflammatory compounds, peptides, postbiotic metabolites	ROS-, pH-, temperature-, or enzyme-responsive carriers combined with an appropriate base delivery platform	Predominantly formulation, ex vivo, and preclinical evidence; clinical validation remains limited	[223,224,225,226]

**Table 7:** An integrative comparison of microbiome-directed and multifunctional bioactive strategies for skin aging: mechanisms, evidence maturity, translational limitations, and relevant delivery approaches.

Bioactive Strategy or Delivery Approach	Representative Examples	Main Mechanisms Relevant to Skin Aging	Highest Available Evidence	Main Advantage	Key Limitation	Current Translational Status	Relevant Delivery Approach	Key References
Probiotics, postbiotics, and synbiotics	*Lactobacillus plantarum* HY7714; *Bifidobacterium breve* M-16V; heat-treated *Latilactobacillus sakei* KABP-065; bacterial lysates	Microbiome modulation, barrier support, inflammatory regulation, oxidative stress reduction, gut–skin signaling	Selected controlled human studies; additional preclinical and in vitro evidence	Multidimensional activity targeting microbial, barrier, and inflammatory pathways	Effects are strain-, dose-, formulation-, route-, and population-specific	Early clinical translation; selected interventions have controlled human evidence	Oral formulations; topical lysates or postbiotics; encapsulated microbial-derived systems	[12,128,129,130,131,132,133,134,135,136,137,138,139,140,141,142,143,144]
Phytoestrogens and polyphenols	Genistein, daidzein, resveratrol, curcumin, epigallocatechin gallate	Estrogen-responsive signaling, antioxidant activity, MMP modulation, ECM support, microbiome-mediated metabolite effects	Limited controlled human evidence; substantial mechanistic support	Multi-target activity relevant to oxidative, endocrine, inflammatory, and ECM pathways	Variable bioavailability, hormonal context, metabolism, and study heterogeneity	Moderate translational maturity, but clinical effects remain compound- and formulation-specific	Liposomes, NLCs, nanoemulsions, polymeric nanoparticles	[145,146,147,148,149,150,151,152,153]
Bioactive peptides and collagen-derived compounds	GHK-Cu, Pal-KTTKS, GEKG, hydrolyzed collagen peptides	Fibroblast stimulation, collagen synthesis, ECM remodeling, MMP modulation, hydration support	Controlled human evidence for selected collagen peptides and signal peptides; product-specific evidence for others	Direct targeting of dermal matrix biology and fibroblast-related pathways	Variable study quality, product sponsorship, limited independent replication, and uncertain dermal delivery after topical use	Moderate clinical translation for selected oral and topical products	Liposomes, polymeric carriers, ultra-deformable vesicles, microneedle-assisted systems for deeper targets	[154,155,156,157,158,159,160,161,162]
Antioxidants and mitochondrial protectors	Vitamin C, niacinamide, coenzyme Q10, vitamin E, astaxanthin, ergothioneine, α-lipoic acid	ROS reduction, redox support, mitochondrial protection, photoprotection, collagen preservation	Heterogeneous evidence; selected controlled human studies and extensive mechanistic support	Broad relevance across oxidative stress, mitochondrial dysfunction, photoaging, and barrier impairment	Instability, oxidation, low solubility, formulation-dependent penetration, and variable clinical outcomes	Moderate translational maturity; strongest evidence is compound-specific	Conventional liposomes, SLNs, NLCs, nanoemulsions	[163,164,165,166,167,168,169,170,171,172]
Adaptogens and metabolic modulators	*Withania somnifera*, melatonin, vitamin D, berberine	Stress axis modulation, circadian regulation, metabolic control, endocrine–immune signaling, oxidative stress reduction	Predominantly systemic, preclinical, or exploratory skin aging evidence	Targets upstream systemic contributors to inflammaging, metabolic dysfunction, and impaired repair	Limited direct evidence for clinically meaningful skin aging outcomes; endocrine interactions require caution	Early translational stage for cutaneous anti-aging use	Mainly oral/systemic strategies; selected topical formulations for melatonin or antioxidant-related compounds	[173,174,175,176,177,178,179,180,181,182,183,184,185]
Conventional lipid-based nanocarriers	Conventional liposomes, SLNs, NLCs, nanoemulsions	Improve stability, superficial retention, controlled release, and local delivery of lipophilic or oxidation-prone compounds	Predominantly ex vivo, preclinical, and formulation evidence; limited product-specific human data	High formulation versatility and relatively favorable scalability for surface- and barrier-directed applications	Enhanced retention or penetration does not establish clinical anti-aging efficacy or reliable dermal delivery	Translational platform stage; clinical benefit remains incompletely established	Primarily skin surface, stratum corneum, upper viable epidermis, and follicular openings	[186,187,201,202,203,204,205,206,215,216,217,218,219]
Advanced and assisted-delivery systems	Transfersomes, ethosomes, transethosomes, polymeric nanoparticles, microneedle-assisted systems, stimuli-responsive carriers	Enhanced viable epidermal delivery, follicular targeting, triggered release, and potentially assisted superficial dermal access	Mainly formulation, ex vivo, and preclinical evidence	May improve compartment-specific delivery for selected cargos	Skin penetration is formulation- and barrier status-dependent; safety, reproducibility, irritation, and systemic exposure require evaluation	Early translational stage; robust controlled clinical evidence remains sparse	Viable epidermis, follicular unit, or superficial dermis with validated assisted delivery	[191,192,193,194,195,196,197,207,212,213,223,224,225,226,227]

## Data Availability

No new data were created or analyzed in this study. Data sharing is not applicable to this article.

## Abbreviations

The following abbreviations are used in this manuscript:

ACTH	Adrenocorticotropic hormone
AGE(s)	Advanced glycation end product(s)
AMPK	AMP-activated protein kinase
AP-1	Activator protein 1
ATP	Adenosine triphosphate
CLSM	Confocal laser scanning microscopy
CoQ10	Coenzyme Q10
CRH	Corticotropin-releasing hormone
DHEA	Dehydroepiandrosterone
DNA	Deoxyribonucleic acid
DSC	Differential scanning calorimetry
E2	17β-estradiol
ECM	Extracellular matrix
EGCG	Epigallocatechin gallate
ERα	Estrogen receptor alpha
ERβ	Estrogen receptor beta
FFAs	Free fatty acids
FTIR	Fourier-transform infrared spectroscopy
GABA	Gamma-aminobutyric acid
GHK-Cu	Glycyl-L-histidyl-L-lysine copper complex
GLUT4	Glucose transporter type 4
GPER	G protein-coupled estrogen receptor
HA	Hyaluronic acid
HDAC	Histone deacetylase
HPA	Hypothalamic–pituitary–adrenal
HPR	Hydroxypinacolone retinoate
Hsp70	Heat shock protein 70
IGF-1	Insulin-like growth factor-1
IL	Interleukin
IL-6	Interleukin 6
IRS	Insulin Receptor Substrate
JNK1	c-Jun N-terminal kinase 1
MAPK	Mitogen-activated protein kinase
MMP(s)	Matrix metalloproteinase(s)
mTOR	Mechanistic target of rapamycin
NAD+	Nicotinamide adenine dinucleotide
NADP+	Nicotinamide adenine dinucleotide phosphate
NF-κB	Nuclear factor kappa B
NLC	Nanostructured lipid carrier
NRF2	Nuclear factor erythroid 2-related factor 2
OCTN1/SLC22A4	Organic cation transporter novel type 1/solute carrier family 22 member 4
PCL	Polycaprolactone
PGC-1α	Peroxisome proliferator-activated receptor gamma coactivator 1-alpha
PI3K	Phosphoinositide 3-kinase
PLA	Polylactic acid
PLGA	Poly (lactic-co-glycolic acid)
PLE	Polypodium leucotomos extract
PNIPAM	Poly(N-isopropylacrylamide)
RGD	Arginine-glycine-aspartic acid
ROS	Reactive oxygen species
SASP	Senescence-associated secretory phenotype
SCFA(s)	Short-chain fatty acid(s)
SLN	Solid lipid nanoparticle
SPF	Sun protection factor
TEWL	Transepidermal water loss
TGF-β	Transforming growth factor-beta
Th	T helper
TNF-α	Tumor necrosis factor-alpha
UV	Ultraviolet
UVA	Ultraviolet A
UVB	Ultraviolet B
VISIA	Facial skin analysis imaging system
